# Loss of filamentous actin, tight junction protein expression, and paracellular barrier integrity in frataxin-deficient human brain microvascular endothelial cells—implications for blood-brain barrier physiology in Friedreich’s ataxia

**DOI:** 10.3389/fmolb.2023.1299201

**Published:** 2024-01-11

**Authors:** Frances M. Smith, Daniel J. Kosman

**Affiliations:** Jacobs School of Medicine and Biomedical Sciences, Department of Biochemistry, The State University of New York at Buffalo, Buffalo, NY, United States

**Keywords:** blood-brain barrier, permeability, filamentous actin, tight junction, transendothelial electrical resistance, frataxin, Friedreich’s ataxia

## Abstract

**Introduction:** Friedreich’s Ataxia (FRDA) is the most prevalent inherited ataxia. FRDA results from loss of Frataxin (FXN), an essential mitochondrial iron trafficking protein. FRDA starts with an early burst of neurodegeneration of the dorsal root ganglion and cerebellar dentate nuclei, followed by progressive brain iron accumulation in the latter. End stage disease includes cardiac fibrosis that contributes to hypertrophic cardiomyopathy. The microvasculature plays an essential barrier role in both brain and heart homeostasis, thus an investigation of this tissue system in FRDA is essential to the delineation of the cellular dysfunction in this genetic disorder. Previous reports have identified cytoskeletal alterations in non-barrier forming FRDA cell models, but physiological consequences are limited.

**Methods:** We investigated brain microvascular endothelial cell integrity in FRDA in a model of the blood-brain barrier (BBB). We have knocked down FXN in immortalized human brain microvascular endothelial cells (hBMVEC), which compose the microcapillaries of the BBB, by using shRNA. We confirmed known cellular pathophysiologies of FXN-knockdown including decreased energy metabolism, markers of oxidative stress, and increased cell size.

**Results:** We investigated cytoskeletal architecture, identifying decreased filamentous actin and Occludin and Claudin-5 tight junction protein expression in shFXN hBMVECs. This was consistent with decreased transendothelial electrical resistance (TEER) and increased paracellular tracer flux during early barrier formation. shFXN hBMVEC start with only 67% barrier integrity of the controls, and flux a paracellular tracer at 800% of physiological levels.

**Discussion:** We identified that insufficient FXN levels in the hBMVEC BBB model causes changes in cytoskeletal architecture and tight junction protein abundance, co-incident with increased barrier permeability. Changes in the integrity of the BBB may be related to patient brain iron accumulation, neuroinflammation, neurodegeneration, and stroke. Furthermore, our findings implicate other barrier cells, *e.g.,* the cardiac microvasculature, loci of disease pathology in FRDA.

## Introduction

Friedreich’s Ataxia (FRDA) is the most prevalent inherited ataxia, affecting ∼1:50,000 US citizens (Indelicato et al., 2020). FRDA is diagnosed around the age of 15, with ∼20 years of degenerating quality of life before patient death around the age of 37 often due to cardiac fibrosis contributory to hypertrophic cardiomyopathy (Payne and Wagner, 2012; Patel et al., 2016). FRDA is caused by GAA expansion repeats within the first intron of the Frataxin (*FXN*) gene, leading to replication stress and transcriptional repression which thereby decreases protein production (Campuzano et al., 1996; Campuzano et al., 1997). In mice, the homozygous *fxn*/*fxn* ^*-*^ genotype is embryonic lethal, reinforcing the essential function of FXN in cellular physiology (Cossée et al., 2000). FXN is a protein localized to the mitochondrial matrix space, assisting in iron incorporation into iron-sulfur clusters (ISC) and heme centers (Colin et al., 2013). ISC and iron-porphyrin prosthetic groups support diverse enzymatic functions including DNA repair, iron homeostatic dynamics, and importantly, energy metabolism. Thus, FRDA is a metabolic disease, as patients experience severe mitochondrial defects, including decreased electron transport chain (ETC) complex expression, depressed oxidative energy metabolism, diabetes mellitus type-II, and buildup of blood lactate (Finocchiaro et al., 1988; Heidari et al., 2009; Van Houten and Wang, 2012; Garg et al., 2017; Smith and Kosman, 2020; Zhao et al., 2020).

The FRDA patient first experiences an early burst of neurodegeneration and atrophy of the cerebellar dentate nuclei (CDN) and dorsal root ganglion (DRG); progressive iron accumulation occurs in the CDN over the progression of disease (Koeppen et al., 2007; Ward et al., 2019). Neurodegeneration in these high-velocity cognitive processing centers confers loss of sensory-motor function and limb control, ultimately causing ataxic gait.

The CDN is suggested to be susceptible to iron accumulation due to its naturally-enriched iron content, but FRDA brain-iron pathology remains poorly understood (Koeppen et al., 2007). That the brain iron accumulation (BIA) occurs *longitudinally* with disease progression has contributed to our hypothesis that BIA in FRDA is an active deposition process, supported by iron flux through (*transcellularly*) or between (*paracellularly*) brain microvascular endothelial cells (Ward et al., 2019).

The loss of FXN’s iron chaperoning capacity in FRDA cells results in intra-mitochondrial iron accumulation, a process that generates reactive oxygen species (ROS). This is further compounded by loss of antioxidant signaling via decreased activity of Nrf2, a major antioxidant transcription factor (Bulteau et al., 2004; Shan et al., 2013; Smith and Kosman, 2020). In addition, levels of free glutathione, a redox regulator, is found to be significantly depleted in FRDA patient-isolated fibroblasts. This is co-incident with glutathionylation of actin monomers, a process now known to be inhibitory of actin filament formation in both the rate and extent of polymerization (Dalle-Donne et al., 2003; Pastore et al., 2003). This is an indirect modulation of the cytoskeleton in FRDA, however, there is direct modulation as well. *PIP5K-1β* is the gene directly upstream of FXN, which is silenced via transcriptional repression in FRDA due to the replication stress caused by FXN’s expansion repeats (Bayot et al., 2013). PIP5K-1β is functionally linked to actin cytoskeletal dynamics, and its loss in FRDA patient fibroblasts leads to altered cell spreading and actin remodeling dynamics (Van den Bout and Divecha, 2009; Bayot et al., 2013).

The findings of increased actin glutathionylation and altered *PIP5K-1β-*based actin dynamics were first reported in fibroblasts, however the integrity of the cytoskeleton in other cell models is lacking. This is of particular importance in cells that are highly reliant on cytoskeletal architecture and integrity for proper function. These include barrier-forming cells, which are reliant on F-actin for tethering of the transmembrane adhesion proteins, *i.e.*, tight junctions. We therefore hypothesize that a degradation of the actin network in FRDA compromises tight junction integrity between adjacent cells of the vasculature, structures essential to the barrier function of these cells. We are particularly interested in human brain microvascular endothelial cells (hBMVEC), which compose the blood-brain barrier (BBB), and their alteration in FRDA.

The BBB is an impermeable barrier due to apically-most facing tight junctions (TJs), maintaining separation of the circulation and the brain interstitium. The BBB is selectively permeable via uptake and efflux transporters and vesicular trafficking, but paracellularly impermeable to molecules exceeding ∼400 Da (Gawdi and Emmady, 2020). Actin filaments anchor the transmembrane TJ proteins (claudins and occludins) via the cytosolic protein zona occludens (ZO) family members (Odenwald et al., 2017; Van Itallie et al., 2017). The transmembrane TJ proteins then form homodimers with their same family members on adjacent cells to maintain paracellular impermeability (Lassowski et al., 2006). While FXN expression is high in brain homogenate, the presence or role of FXN in brain microvascular endothelial cells has not been investigated. We sought to extend FRDA research in this highly specialized and essential microvascular system.

BBB research is limited in the FRDA literature, and examination of barrier function could provide therapeutic targets for FRDA neurophysiology. We have hypothesized that FXN-deficient hBMVEC have altered cytoskeletal and tight junction architecture that contributes to paracellular permeability. Here we examine the vascular integrity in FXN-knockdown hBMVEC. Following shRNA-mediated FXN knockdown, our hBMVEC display a loss of total and membranous filamentous actin, decreased tight junction protein expression, and increased paracellular permeability in a transwell barrier model system. Our findings suggest that investigation of vascular integrity in FRDA patients likely would open new therapeutic windows addressing brain solute influx and neuropathology in this disease. Furthermore, our results may be translational to other vascular barrier systems, for instance of the heart, in relation to cardiac pathology.

## Methods

### Reagents

All chemical reagents are obtained from Sigma Aldrich unless otherwise stated. Lyophilized compounds are solubilized per manufacturer instructions. All protocols are performed per manufacturer instructions unless otherwise stated.

### Antibodies

Primary: Rabbit α-FXN (ThermoFisher #PA5-13411, used at 1:500), Mouse α-TATA-binding protein (ThermoFisher #49-1036, used at 3.2 μg/mL), Rabbit α-Nrf2 (AbClonal #A21176 used at 1:750), Rabbit α-claudin-5 (Abcam #ab131259 used at 1:1,000), Rabbit α-occludin (Abclonal #A2601, used at 1:1,000), Rabbit α-ZO1 (Abclonal #A0659, used at 1:5,000), Goat α-Transferrin Receptor (R&D Systems #AF2474, used at 0.25 μg/mL), Rabbit α-Ferroportin (Novus #21502SS, used at 1:1,000), and Rabbit α-β-actin (Cell Signaling Technologies 8457, used at 1:5,000). All primary antibodies were diluted in 1% milk-TBST.

Secondary: Donkey α-goat:647 (Thermo Fisher #A21447, used at 1:1,000), Donkey α-rabbit:488 (Thermo Fisher #A21206, used at 1,1:000), Goat α-mouse:HRP (Novus Biologicals NBP2-31347H, used at 1:5,000), and Goat α-rabbit:HRP (Cell Signaling Technologies #7074, used at 1:5000). All secondary antibodies were diluted in 3% milk-TBST. All chemiluminescent antibodies were activated using a 1–5 min was of ECL reagents (Clarity Max, Biorad). All blot imaging was performed on the ChemiDoc Illuminator (Biorad).

### qPCR primers

Primer sequences are represented in Supplementary Table S1.

### Cell culture

Wild-type human brain microvascular endothelial cells (hBMVEC) are an immortalized cell line, a gracious gift from Dr. Supriya Mahajan (University at Buffalo). Verification of proper cell behavior is provided (Franke et al., 1999; Eigenmann et al., 2013). hBMVEC were cultured in RPMI, +84 μM Penicillin, +91 μM Streptomycin, +10% FBS (Gibco), and +10% Nuserum (Corning), passaged at confluency and used between passage numbers 7 and 15 in experiments. Cells were housed in a 37°C incubator, 5% CO_2_ injection, and constant humidity.

### Knockdown

shRNA was generated against FXN using the commercial pGIPZ system along with their empty vector (EV) backbone control (Horizon Discovery). Briefly, glycerol stocks were plated and selected on Ampicillin-containing LB agar at 30°C overnight. Single colonies were selected and grown in liquid culture for MaxiPrep (Omega EZNA) at 37°C, shaking overnight. Plasmid DNA for shFXN, EV, and psPAX2 and pMD2.G packaging systems (gracious gifts from Didier Trono: Addgene plasmids 12,260 and 12,259 respectively) were eluted into TE elution buffer, and concentration and purity were quantified via NanodropOne (Invitrogen). HEK293T cells were plated at 5 million cells/10 cm dish in DMEM +10% FBS and let attach overnight. The following morning, 15 µg psPAX2, 6 µg pMD2.G, and 20 µg of each shRNA or EV plasmid DNA were combined with CaCl_2_ and HBS for Calcium Phosphate transfection as described (Dull et al., 1998). Briefly, the DNA mixture was incubated at room temperature for 15 min then added dropwise to the HEK293T cells. Media was changed 6 h following transfection to normal growth media. Virus-containing media was collected at 48- and 72-h following transfection, filtered through 0.22 µM PES filter, and concentrated using a sucrose-gradient ultracentrifugation.

The viral pellet was solubilized in RPMI +10% FBS, 10%Nuserum. 20 μL of each virus (shFXN and EV) + 5 μg/mL Polybrene was added to wild-type hBMVEC for 6 h, then replaced with growth media. 2 days following transfection, shRNA-containing cells were selected for in growth media +0.75 μg/mL Puromycin. Cell populations were allowed to grow back to confluency with minimal media changes. Proper shRNA expression was verified by the presence of GFP expression using the ZOE fluorescent imager. shRNA-integrated cells are continued in culture under selective pressure until plated for an experiment, at which media was replaced with normal RPMI.

### Reverse transcription quantitative polymerase chain reaction (RT-qPCR)

hBMVEC were lysed in Trizol (Ambion Life Technologies) and RNA was isolated using Direct-Zol MiniPrep spin columns (Zymogen) per manufacturer instructions with additional RNAse-OUT (Invitrogen) treatment during DNA digestion. RNA eluted in RNAse-free water was quantified for both concentration and purity using the NanodropOne (Invitrogen). 400 ng of RNA was reverse transcribed using qScript (QuantaBio), of which 20 ng each was loaded into a qPCR plate with a master mix of 5 µL iTaq SYBR Green (Biorad) and 500 nM each forward and reverse primers for; FXN, TfR, Fpn, Catalase, SOD1, SOD2, GLRX1, GLRX2, Nrf2, Claudin-5, Occludin, ZO-1, β2M, and β-actin. The absence of gDNA was confirmed by cycling equimolar concentrations of RNA that had not been reverse transcribed. Differential abundance of transcript was assessed using the -ΔΔCt method of quantitation with β2M as the housekeeping gene, normalized to the values of the EVEC control.

### Western blotting - FXN, TfR, Fpn, and TBP

hBMVEC were lysed in RIPA buffer containing 4x protease inhibitor and kept on ice. Lysates were centrifuged at 4°C for 15 min at 13,000 RPM. Supernatant was separated from cell debris and protein quantified using the BCA method at 562 nm absorbance (Thermo Scientific). 20–25 μg of protein was added to 1x Laemli-buffer containing 150 mM dithiothreitol (DTT) for denaturation at 37°C for 30 min. Lysates were electrophoresed on a 12% bis-tris Bolt gel (ThermoFisher) alongside either 2.5 µL Magic Mark protein molecular weight marker (Thermo Fisher) or 3.5 μL All Blue standard protein ladder (BioRad) at 140 V. The gels were then transferred to a water-activated nitrocellulose membrane at 1.3 A to 25 V for 7 min (BioRad TurboBlotter, Mixed Molecular Weight setting). Membranes were blocked in Every Blot Blocking Buffer (Biorad) for 10 min, rocking at room temperature. All primary antibodies were diluted in 1% milk and TBST, and rocked over the solution at 4°C overnight. The next day, the membrane was washed thrice in 10-min washes of 1x TBST, followed by a 1-h room temperature incubation of secondary antibodies in 3% milk and TBST. Rabbit αFXN was followed by αRabbit:HRP and Rabbit αFpn and Goat αTfR were followed with Donkey αRabbit:488 and Donkey αGoat:647, respectively. FXN was normalized to mouse α-TATA-binding protein (TBP) followed by αMouse:HRP. TfR, and Fpn were normalized to the band intensity of rabbit α-TBP followed by αRabbit:HRP. Bands were quantified using densitometry (ImageLab, BioRad), data is represented as band of interest normalized to its control, further normalized to the EVEC controls.

### Stain-free Western blotting–Claudin-5, occludin, ZO-1, Nrf2, and β-actin

Lysates were prepared as described above, and ran on a 4%–20% Stain-free gel (BioRad) alongside 3.5 µL All Blue protein standard (Biorad). Following electrophoresis, the gel was UV-illuminated for 1 min using the GelDoc (Biorad). The gel was transferred to an ethanol-activated PVDF membrane as above and UV-excited again to quantify total protein transferred. Primary and secondary incubations followed as above. Rabbit αClaudin-5 was followed by αRabbit:HRP and ECL reagents. Rabbit α-Occludin and Rabbit α-ZO-1 were followed by αRabbit:647 before imaging. Rabbit α-β-actin, Rabbit α-Claudin-5, and Rabbit α-Nrf2 were followed by αRabbit:HRP. Densitometric analysis was used to normalize band intensity to total protein transferred, then further normalized to values of the EVEC controls.

### Agilent Seahorse metabolic analysis

Energy metabolism was assessed via Seahorse Mito Stress Test (Agilent). An XF96-well Seahorse plate was coated for 5 minutes in 0.1 mg/mL Poly-D-Lysine, 0.01 N HCl, followed by two washes in water and one wash in PBS. Cells were seeded at 8,800 cells per well in a 96-well Seahorse plate in normal growth RPMI containing serum. 24-h prior to the assay, the sensor cartridge was equilibrated in sterile water overnight at 37°C without CO_2_, and changed to calibrant for 1 hour in the same incubation conditions. 36 h after plating, cell media was removed and replaced with 200 µL Agilent assay media (RPMI without phenol red, +10 mM glucose, +1 mM pyruvate, and +2 mM glutamine) for 1 hour, incubating at 37°C without CO_2_. The media was aspirated and again replaced with 180 µL assay media. The sensor cartridge was loaded so that cells were treated at a final concentration of 2.5 µM Oligomycin in the A injection port, 1 µM FCCP in the B injection port, and a cocktail of 0.5 µM each Rotenone and Antimycin-A in port C per manufacturer instructions. Each experiment was preceded by the sensor calibration. Following the assay, the wells were washed once in PBS, and lysed using NP-40 based buffer. Protein content as quantified by BCA was used for normalization.

### MitoBright and Mitochondrial Morphology analysis

hBMVEC were seeded on sterile coverslips and allowed to reach 50% confluence. The protocol was followed per manufacturer instructions. Briefly, cells were treated with mitobright at a concentration of 1:1,000 with 0.7 μg/mL Hoechst-33342 in culture media for 15 min at 37°C. The coverslips were washed twice in PBS before fixing for 10 min in 3.7% paraformaldehyde, 4% sucrose in PBS at room temperature. The coverslips were washed twice again before mounting to glass slides with prolong-gold antifade mounting media (ThermoFisher). Images were acquired using the ×63 oil immersion objective of the Leica DMi8 inverted microscope. At least 6 images were taken per coverslip for three individual experiments. The mitochondrial network of individual cells was analyzed using the Mitochondrial Morphology macro on ImageJ (NIH, Bethesda, MD) per author instructions (Dagda et al., 2009).

### Phalloidin-Texas Red—total actin staining

hBMVEC were seeded on sterile coverslips and allowed to reach 50% confluence before fixation in 3.7% paraformaldehyde, 4% sucrose for 10 min at room temperature, followed by blocking in 0.1% BSA and 0.01% Tween-20 for 1 h at room temperature. Phalloidin-Texas Red (ThermoFisher) was diluted at 1:400 in PBS per manufacturer instructions with 0.7 ug/mL Hoechst-33342 for 45 min at room temperature. The coverslips were then washed thrice in PBS and mounted as previously described. Images were acquired using the ×63 oil immersion objective of the Leica DMi8 inverted microscope. Total polymerized actin was quantified as the integrated density value of phalloidin divided by that of the Hoechst channel acquired via ImageJ, then normalized to EVEC controls.

### Phalloidin-Texas Red—membrane-bound and cortical actin staining

Peripheral actin was quantified by drawing a line of 6.405 µm across cell membranes in ImageJ. The middle of the line was placed at the extracellular-facing start of the plasma membrane. Two to five membrane regions of interest were quantified per cell per image and tabulated as a histogram as previously described using the “Plot Profile” function (Vosolsobě et al., 2017; Vosolsobě et al., 2018; Steimle et al., 2022). Individual traces were combined and analyzed (Prism5) to obtain the average trace per condition. The peak of the line is defined by the middle of the original line drawn, or the start of the membrane. The neighboring 300 nm on either side of the peak was assigned as the cortical actin ring (Svitkina, 2020). Representative images are cropped from their original size, without manipulating the size of the scale bar.

### Transendothelial electrical resistance (TEER) measurements

hBMVEC were seeded apically in 1 µm pore size, 24-well transwell inserts (Greiner BioOne) with normal growth media in both chambers and then polarized with serum-free RPMI containing 300 nM sodium selenite and 5 μg/mL insulin in the basal compartment 8-h post plating. TEER measurements were acquired using the Endohm-6G (World Precision Instruments) connected to the MillCell-ERS2 voltammeter (Millipore). Ohm values collected at 24, 48, 72 and 96-h post-plating were subtracted from an identical blank transwell with no cells. Blank-corrected resistance values were then multiplied by the growth area of the 24-well transwells (0.336 cm^2^). At 96-h post plating, apical media aliquots were removed and assayed for lactate dehydrogenase (LDH) secretion (Cytox-96, ThermoFisher) to measure cell death. To account for any seeding differences between passage number or cell number seeded between independent experiments, the cell monolayer was lysed in 200 µL of NP-40 and quantified for protein content using the BCA.

### Lucifer Yellow paracellular tracer flux

hBMVEC were seeded apically in 1 µm 24-well transwell inserts (Greiner BioOne) with growth media in both chambers, then polarized with serum-free RPMI containing 300 nM sodium selenite and 5 μg/mL insulin in the basal compartment 8-h post plating. At 24-, 48-, 72-, and 96-h in culture hours in culture, the transwells were incubated apically with growth media containing 50 µM Lucifer Yellow (LY). Basal media aliquots were removed after 45-min, quantified for fluorescence at 428 nm excitation, 536 nm emission, and calculated against a standard curve to generate micromolar of LY fluxed. Apical media aliquots were sampled prior to LY-media changes and quantified for cell death using LDH secretion (Cytox-96, ThermoFisher).

### Statistics

Prism 5 (GraphPad) was used for statistical analyses and their graphical representation. Datapoints were considered outliers if falling outside of the range of two standard deviations from the mean, and excluded from analysis. All datasets comparing Empty Vector Endothelial Cell (EVEC) control and shFXN were analyzed by Student’s t-test, all with a confidence interval at 95%. The F-test was performed to test if the sample groups had significantly different standard deviations. If true, the Welch’s correction was used, also at a confidence interval of 95%. Experiments performed on a timed basis, including TEER and Lucifer Yellow flux were analyzed using two-way ANOVA at a confidence interval of 95%.

## Results

### FXN is knocked down via shRNA in hBMVEC

We used lentiviral-mediated shRNA knockdown of FXN in our hBMVEC due to the stable and long-term transfection efficiency, generation of non-clonal heterogeneous cell populations, and retention of residual amounts of protein to prevent lethality of frataxin-*knockout.* Indeed, many *in vitro* FRDA studies use similar methods of knock*down* to avoid the lethality of a knock*out* system, e.g., ∼60% FXN protein expression was observed in a shFXN neuronal model (Rodríguez-Pascau et al., 2020; Britti et al., 2018; D’Oria et al., 2013; Franco et al., 2012).

Neither the presence nor the role of FXN has been investigated in hBMVEC. The lentiviral-mediated shRNA targeting FXN used in our *in vitro* knockdown model was compared to the empty-vector backbone control plasmid (Horizon Discoveries). Each shRNA plasmid was packaged into the second-generation lentiviral enveloping systems PMD2.G and psPAX2 in HEK293T cells, and viral particles were transfected into Wild-Type hBMVEC using polybrene. All experiments using the shFXN hBMVEC were compared against the *E*mpty *V*ector *E*ndothelial *C*ell (EVEC) control. Integration of the shRNA was confirmed by GFP expression, which was absent in hBMVEC controls (Supplementary Figure S1).

**FIGURE 1:**
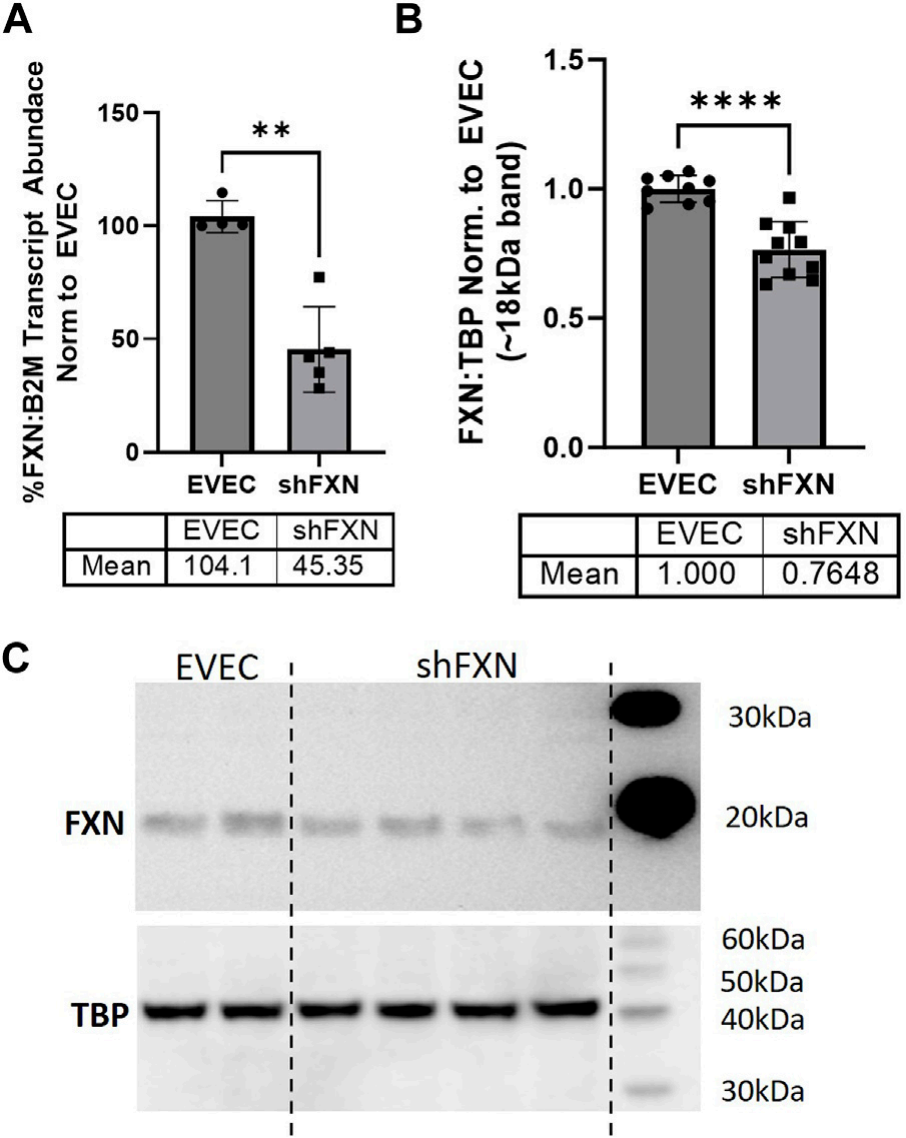
Lentiviral-mediated short-hairpin RNA (shRNA) sufficiently knocks-down frataxin (FXN). **(A)** RNA was reverse transcribed and amplified for FXN and Beta-2-microglobulin (B2M) as a housekeeping control, transcript abundance was quantified using the ΔΔCt method normalized to the empty vector endothelial cells (EVEC). **(B)** Total protein lysates were run on a bis-tris SDS-PAGE, transferred to nitrocellulose, and probed for FXN and Tata-Binding Protein (TBP) as a housekeeping gene. FXN protein expression is normalized to that of TBP as quantified by densitometry, represented as normalized to pooled EVEC values. **(C)** Representative blot is shown. Student’s t-test α = 0.05. **p < 0.01, ****p < 0.0001. **(A)** EVEC; *n* = 4 shFXN; *n* = 6. **(B)** EVEC; *n* = 9, and shFXN2; *n* = 10.

We quantified FXN knockdown using RT-qPCR amplifying the FXN transcript in shFXN and EVEC lines. Beta-2-Microglobulin (B2M), a subunit of the constitutively expressed cell surface component MHC class II was used as the housekeeping reference gene to avoid using the classical housekeeping genes GAPDH (metabolic enzyme) or β-actin (cytoskeletal protein) that would likely reflect changes in energy metabolism and cytoskeletal physiology, respectively (Jiménez-Munguía et al., 2021). Indeed, shRNA in our system was efficient in knocking down FXN, as we observed ∼45% of the FXN transcript of the EVEC controls (Figure 1A).

We then used Western blotting to determine if FXN protein levels were decreased as well. EVEC and shFXN lysates were electrophoresed on a 12% bis-tris gel and transferred to nitrocellulose to probe for FXN and the housekeeping control TATA-binding protein (TBP). shFXN and EVEC have similar levels of TBP as normalized to total protein/lane, making it a suitable housekeeping control (Supplementary Figure S2). The ∼41 kDa major band (arrowhead) was knockout-validated in HeLa cells (ThermoFisher, 2023). TBP is a transcription factor unlikely to be affected in FRDA, and therefore used for normalization (Guccini et al., 2011; Petrillo et al., 2021). Our shFXN hBMVEC retained 76% of normal FXN levels present in the EVEC control (Figures 1B, C). Our blots displayed the intermediate form of the FXN protein, running ∼18 kDa (Koutnikova et al., 1998; Gordon et al., 1999). The use of this 18 kDa band was validated using a FXN-overexpression HEK293T lysate, in which the strongest band was shown at ∼18 kDa (Supplementary Figure S3). The FXN precursor form is cleaved twice prior to its mitochondrial translocation, however, the mature (13 kDa) form of the protein was not visible in our blots (Guo et al., 2018). In summary, our lentiviral-mediated shRNA knockdown of FXN in hBMVEC resulted in retention of ∼45% of transcript and ∼76% of protein compared to EVEC controls.

### shFXN hBMVEC display a slight alteration in iron homeostasis proteins

**FIGURE 2:**
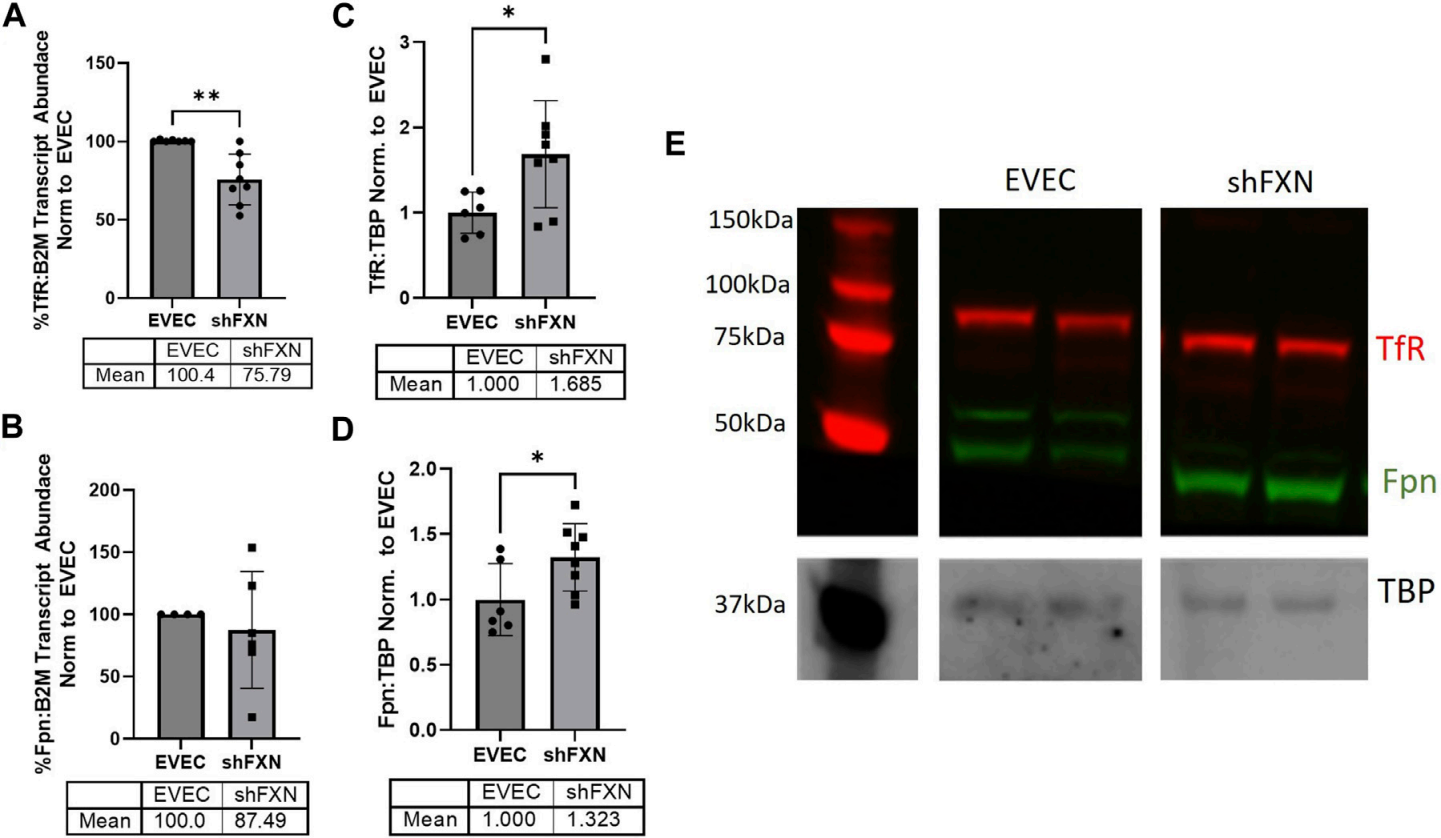
shFXN have altered iron metabolism proteins. RNA was reverse transcribed and amplified for **(A)** Transferrin receptor (TfR), **(B)** Ferroportin (Fpn), and Beta-2-microglobulin (B2M) as a housekeeping control, transcript abundance was quantified using the ΔΔCt method normalized to the empty vector endothelial cells (EVEC). Total protein lysates were run on a 12% bis-tris SDS-PAGE, transferred to nitrocellulose, and probed for **(C)** TfR and **(D)** Fpn protein expression against Tata-Binding Protein (TBP) as a housekeeping gene. Each protein is normalized to that of TBP as quantified by densitometry, normalized to pooled EVEC values. **(E)** Representative blot is shown. TfR is visualized with Alexa-647 (Red), and Fpn with Alexa-488 (Green). Student’s t-test α = 0.05. *p < 0.05, **p < 0.01. **(A)** EVEC; *n* = 2 shFXN; *n* = 3. **(B)** EVEC; *n* = 1, and shFXN2; *n* = 2. **(C–E)** EVEC; *n* = 6 and shFXN; *n* = 8.

FXN-deficient cells have been described to have an iron-starvation phenotype, exhibiting increased iron import via transferrin receptor (TfR), and decreased ferroportin (FPN), the only known mammalian iron export protein (Gabrielli et al., 2012; Alsina et al., 2018). To our surprise, transcriptional analysis of each target showed reduction in the shFXN hBMVEC, retaining ∼76% of TfR and ∼88% of FPN (Figures 2A, B, respectively). However, protein analysis showed an increase in each of these proteins, i.e., 168% for TfR and 132% for FPN (Figures 2C–E). We expected TfR to be increased in line with the iron-starvation hypothesis observed in other FRDA models, but were surprised to see a smaller, albeit significant, increase in FPN. Consistent with the iron starvation hypothesis of stabilization of TfR, our shFXN hBMVEC showed upregulation of TfR protein (Figure 2C). However, we were surprised to see a similar increase in Fpn protein (Figure 2D). Fpn is regulated by three major pathways; 1) degradation via circulating Hepcidin hormone (Charlebois et al., 2022), 2) translational repression via 5′ IRE during iron-replete conditions (Drakesmith et al., 2015), and 3) miR-485-3p targeting the 3′ UTR (Sangokoya et al., 2013). However, since our experiments have been done *in vitro* and lacking paracrine signaling from other cell types, we cannot yet be certain on the regulation of Fpn in our hBMVEC line. Increased Fpn would result in a loss of intracellular Fe^2+^, mitigating some oxidative stress normally characteristic of FXN-deficient cells. Therefore, our model may be missing some normal pathological defects, possibly due to a mild reduction in FXN via shRNA (Figure 1).

### shFXN hBMVEC exhibit increased markers of oxidative stress

**FIGURE 3:**
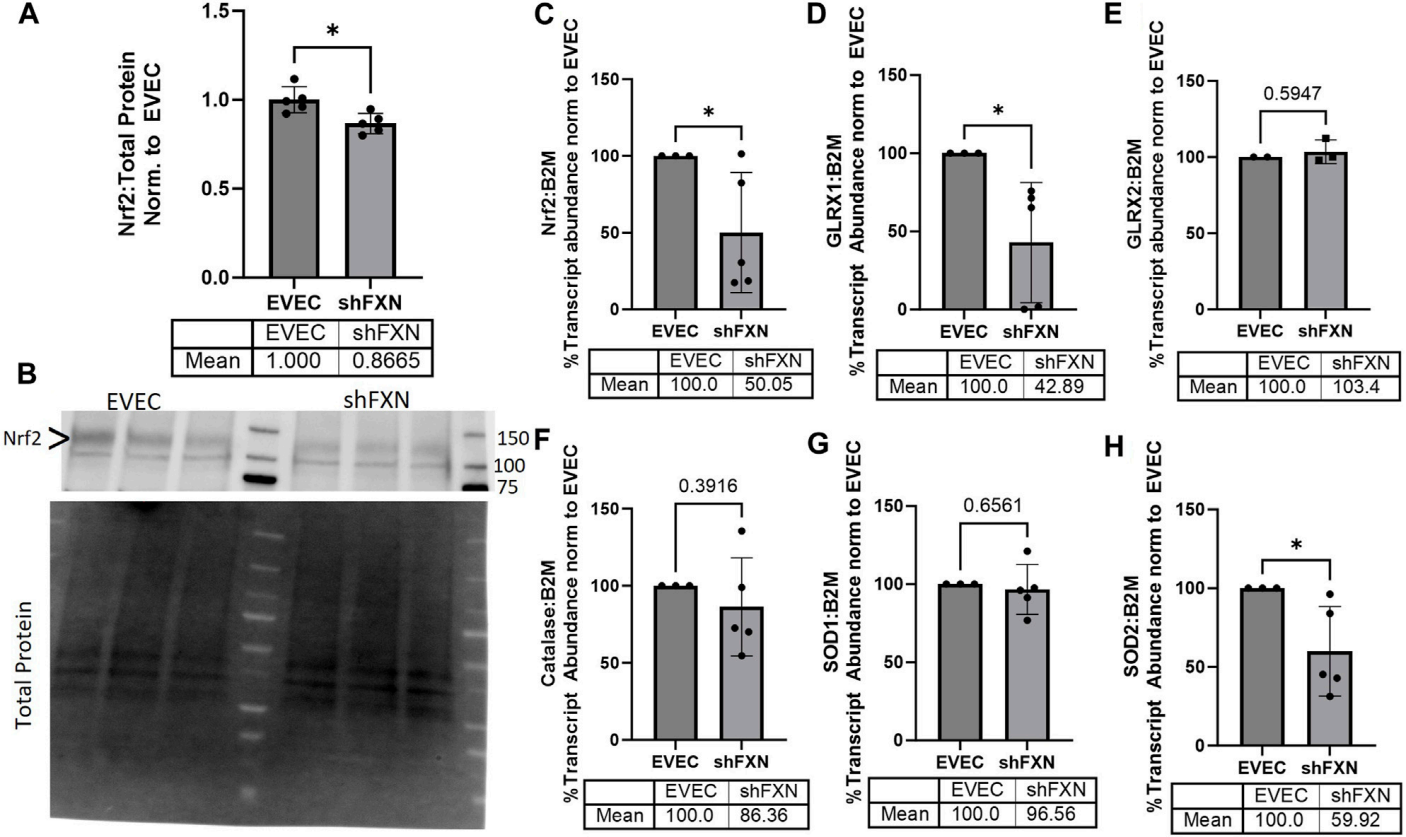
shFXN hBMVEC have a decreased antioxidant capacity. **(A)** Total protein lysates were run on a 4%–20% stain free gel, transferred to PVDF, and probed for Nrf2 against total protein for normalization. Densitometry is further normalized to EVEC controls. **(B)** Representative blot shown. The ∼120 kDa band (represented via arrowhead) is knockout-validated. **(C–H)** RNA was reverse transcribed and amplified for **(C)** Nrf2, **(D)** Glutaredoxin-1 (GLRX1), **(E)** GLRX2, **(F)** Catalase, **(G)** Superoxide Dismutase-1 (SOD1), **(H)** SOD2, and B2M as the housekeeping control. Transcript abundance was quantified using the ΔΔCt method normalized to the empty vector endothelial cells (EVEC). Student’s t-test α = 0.05. *p < 0.05. **(A, B)** EVEC and shFXN; *n* = 5. **(C, D, F–H)** EVEC; *n* = 3. shFXN; *n* = 5. **(E)** EVEC; *n* = 2. shFXN; *n* = 3.

FRDA models display increased oxidative stress downstream of improper iron chaperoning, and this correlates with the increased oxidative actin glutathionylation observed in FRDA patient fibroblasts (Piemonte et al., 2001; Pastore et al., 2003). We therefore sought to quantify markers of redox stress in our shFXN hBMVEC. Nrf2 is a major antioxidant transcription factor which is found decreased in other FRDA models (D’Oria et al., 2013; Paupe et al., 2009; La Rosa et al., 2021; Petrillo et al., 2019). Indeed, we saw that shFXN hBMVEC retained only 86% of EVEC Nrf2 total protein (major band designated via arrowhead) (Figures 3A, B). Again, this is a relatively mild reduction, but this is reflected by the marginal loss of FXN in our shRNA model (Figure 1B). In line with this model, we see a significant decrease in Nrf2 transcript levels in shFXN compared to EVEC controls (Figure 3C).

We further sought to quantify by transcriptional analysis the antioxidant effectors downstream of Nrf2 activation. We analyzed glutaredoxin-1 and -2, the cytosolic and mitochondrial (respectively) de-glutathionylating enzymes. We were particularly interested in GLRX1 due to the known increase in actin glutathionylation in FRDA models (Pastore et al., 2003). Furthermore, we quantified the levels of SOD-1 (cytosolic), SOD-2 (mitochondrial), and catalase, as all three are involved in suppression of reactive oxygen species produced from the reaction of free ferrous iron and molecular oxygen (Smith and Kosman, 2020).

Our observed loss of Nrf2 (Figures 3A–C) was compounded by a 57% loss of the major cytosolic deglutathionylating enzyme GLRX1; the mitochondrial form, GLRX2, was unchanged (Figures 3D, E). Catalase exhibited an insignificant, albeit observable decrease in transcriptional abundance, retaining 86% of physiological levels (Figure 3F). SOD1 was unchanged whereas its mitochondrial form, SOD2 was significantly decreased by 40% in shFXN hBMVEC (Figures 3G–H). Taken together, we saw a significant loss of Nrf2, the major antioxidant regulator, SOD2, a mitochondrial antioxidant, and GLRX1, the cytosolic de-glutathionylating enzyme. This fits with our shFXN hBMVEC model beginning with mitochondrial oxidative stress, leading to actin glutathionylation in the cytosol.

### shFXN hBMVEC have decreased oxidative energy metabolism and ATP production along a with shift to glycolytic energy metabolism

**FIGURE 4:**
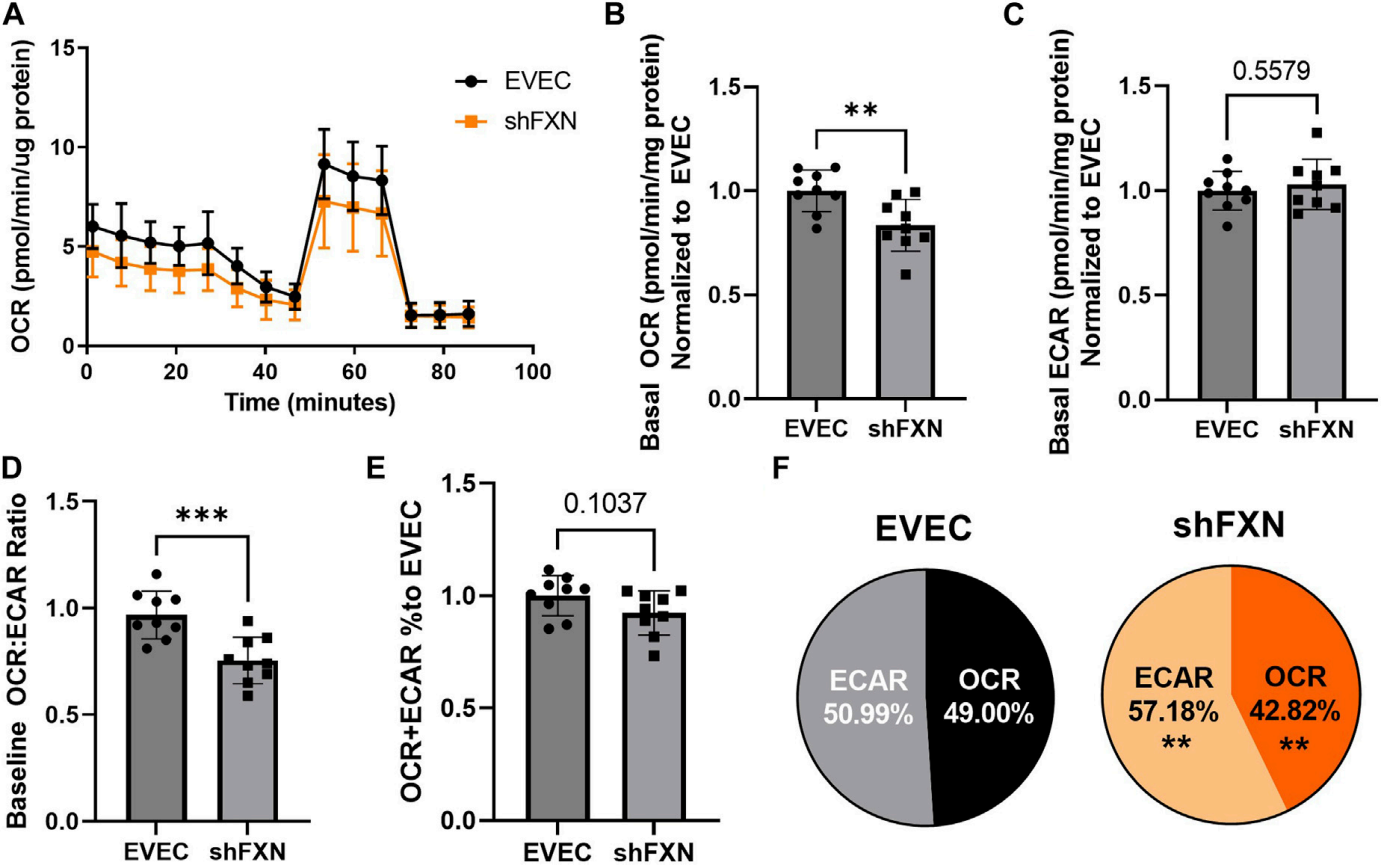
shFXN hBMVEC are deficit in oxidative phosphorylation and compensate by increasing glycolysis. hBMVEC were analyzed in the Agilent Seahorse using the Mito Stress Test. **(A)** Example of oxygen consumption rate (OCR) mito stress test energy trace, EVEC in black and shFXN in orange. Basal levels of Oxygen consumption rate (OCR) **(B)** and extracellular acidification rate (ECAR) **(C)**. **(D)** OCR:ECAR ratio of basal energy metabolism, **(E)** Combined levels of OCR and ECAR metabolic pathways. **(E)** Levels of OCR and ECAR performed in each phenotype represented as % of total metabolism. Each well was lysed and quantified for micrograms of protein to use as a normalization factor, then further normalized to the EVEC controls. Student’s t-test α = 0.05; **p < 0.01, and ***p < 0.001. **(A)**. Lines are averages of three biological replicates each used as a representative graph. **(B–F)** EVEC and shFXN; *n* = 9. Datapoints are representative of nine biological replicates performed in three independent experiments.

Another hallmark of FXN loss is decreased metabolic capacity due to lack of iron incorporation into the electron transport chain (ETC) complexes I-III and aconitase (Rötig et al., 1997; Heidari et al., 2009; Van Houten and Wang, 2012; Martelli and Puccio, 2014). We measured both oxidative phosphorylation and glycolysis in our model using the Agilent Seahorse Mito Stress Test. The data clearly show shFXN hBMVEC (orange) are oxidatively deficient compared to EVEC controls (black) as presented by the oxygen consumption rate (OCR) (Figure 4A). Indeed, shFXN hBMVEC were significantly deficient in oxygen-mediated energy metabolism compared to the EVEC controls as represented by ∼16% reduction in basal OCR (Figure 4B).

We were intrigued to see that the shFXN hBMVEC had a slight, albeit non-significant, increased marker of glycolytic function to ∼103% (extracellular acidification rate (ECAR)) (Figure 4C). Note that the exact influence of FXN knockdown on glycolysis specifically cannot be known as ECAR is representative of not only glycolytic function, but non-mitochondrial respiration as well, as it is influenced by CO_2_ production from the Kreb’s cycle (Mookerjee and Brand, 2015). However, we hypothesize that shFXN hBMVEC are deficient in oxidative modification downstream of FXN loss, and that cells respond with increased non-mitochondrial energy metabolism to make up for this fact.

This shift was indicated by significant decrease in the OCR:ECAR ratio (Figure 4D) with only a marginal decrease in total energy metabolism (Figure 4E). Thus, oxidative phosphorylation was the major pathway affected in shFXN hBMVEC, while glycolysis was significantly upregulated to maintain energy production. This was quantified in a nearly 7% shift in the glycolytic phenotype of shFXN hBMVEC (Figure 4F). These metabolic markers align with the known oxidative phosphorylation pathologies in FRDA models, as well as revealing a related upregulation in glycolytic energy metabolic capacity as well.

### shFXN hBMVEC have more mitochondrial objects and larger cell size

**FIGURE 5:**
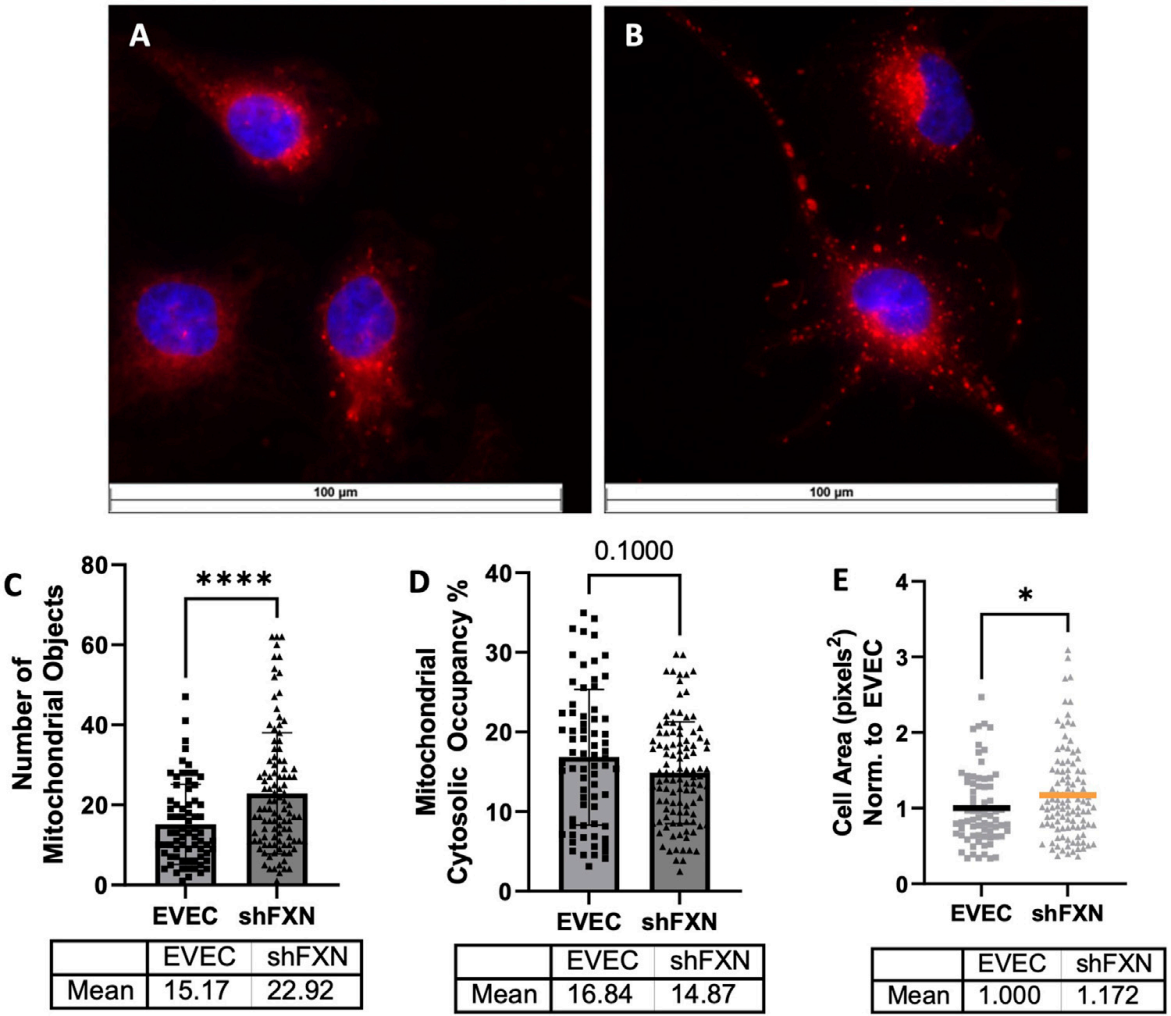
Mitochondrial physiology is altered in shFXN. Representative images are shown in **(A)** EVEC and **(B)** shFXN hBMVEC. **(C)** Mitobright mitochondrial dye is used to determine the number of mitochondrial objects per cell. **(D)** Mitochondrial number is normalized to the cell area to determine the percentage cytosolic occupancy. **(E)** Total cell size is quantified and normalized to EVEC controls. Student’s t-test α = 0.05 *p < 0.05, ****p < 0.0001. **(C)** EVEC; n = 71, shFXN; n = 105. **(D)** EVEC; n = 71, shFXN; n = 106 **(E)**. EVEC; n = 71, shFXN; n = 105 cells analyzed.

Due to FXN’s role in mediating mitochondrial physiology, we sought to determine the effects of FXN knockdown on mitochondrial networking. Aberrant mitochondrial dynamics in both fission and fusion pathways have been reported in FRDA (Chidipi et al., 2021). To investigate mitochondrial networking, we used Mitobright Deep Red (Dojindo) to stain mitochondria, and the Mitochondrial Morphology ImageJ Macro for network analysis in EVEC controls (Figure 5A) and shFXN hBMVEC (Figure 5B) (Dagda et al., 2009). shFXN showed increased levels of mitochondrial number consistent with mitochondrial fission defects in FRDA patient-derived cardiac iPSCs (Chidipi et al., 2021) (Figure 5C). We were surprised to see that the cytosolic occupancy of mitochondria, however, was nearly the same across the cell samples (Figure 5D). This metric quantifies the density ratio of mitochondrial objects normalized to cell size so we were interested in analyzing cell area. Indeed, shFXN hBMVEC were significantly larger than EVEC controls, representing a 17% increase in cell area (Figure 5E). FRDA patient fibroblasts with increased actin glutathionylation exhibited increased cell size, also (Piemonte et al., 2001; Pastore et al., 2003). The observed changes in shFXN cell size led us to investigate potential changes in actin filament abundance and distribution, and changes in barrier function.

### shFXN hBMVEC have decreased levels of polymerized total and peripheral actin

Because actin glutathionylation is increased in FRDA models and is correlated to decreased filamentous (F-actin) formation, we wanted to investigate F-actin formation in our shFXN hBMVEC (Dalle-Donne et al., 2003; Pastore et al., 2003). F-actin is essential in barrier physiology due to its anchorage of tight junction proteins via the scaffolding protein ZO-1. In addition, underlying membranous actin sits the cortical actin ring (CAR), a network of actin fibers that in addition to providing junctional support also increases structural integrity and extracellular matrix adhesion. We wanted to correlate our findings of reduced GLRX1 (Figure 3D) and known actin glutathionylation in patient fibroblasts to a decrease in filamentous actin formation in shFXN hBMVEC (Piemonte et al., 2001; Dalle-Donne et al., 2003; Pastore et al., 2003).

**FIGURE 6:**
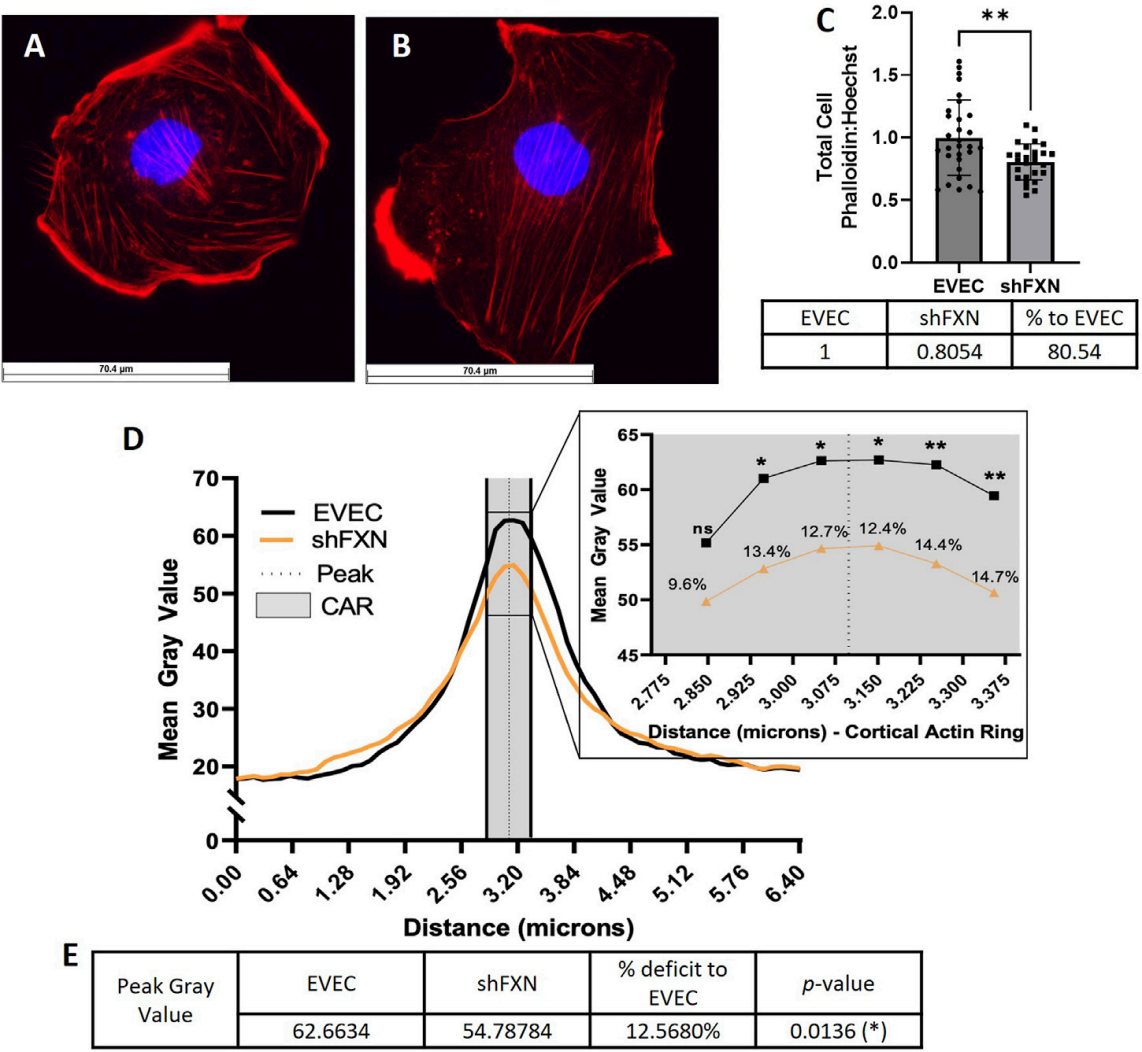
shFXN hBMVEC are deficient in total, peripheral, and cortical filamentous actin. **(A)** EVEC and **(B)** shFXN hBMVEC are incubated with Phalloidin-Texas Red and Hoechst, **(C)** the ratio taken to quantify total filamentous actin per cell, then normalized to EVEC controls. **(D)** 6.4 µm of two to five membranes per cell were quantified for F-actin (gray value) and averaged into a histogram. The membrane interface (peak) is represented by the dotted line. 300nm surrounding the peak was quantified as the cortical actin ring (CAR), as represented by the shaded region. Individual datapoints of the cortical ROI are represented in the inset with significance and the %deficit in F-actin. **(E)** Values and %deficit with statistics for the peak membrane F-actin. Student’s *t*-test α = 0.05; ns = not significant, *p < 0.05, **p < 0.01. Total phalloidin analysis **(C)**: EVEC; *n* = 30, shFXN; *n* = 27. Membranes analyzed **(D, E)**: EVEC; *n* = 191, shFXN; *n* = 222. The images in panels **(A, B)** were cropped from their original size while maintaining the scale of the scale bar.

Using phalloidin-Texas red, a dye which stains only F-actin, we quantified total, membranous, and CAR actin fibers in EVEC (Figure 6A) and shFXN (Figure 6B) hBMVEC. We quantified a significant reduction of total F-actin in shFXN hBMVEC compared to EVEC, representing a nearly 20% F-actin loss (Figure 6C). Differential and disorganized phalloidin staining at the cell membrane was apparent (Figure 6B *versus* 6A), as well, so we quantified pixel intensity of Phalloidin Texas-Red along a 6.4 micron line drawn through the cell membrane (Vosolsobě et al., 2017; Vosolsobě et al., 2018). The line was drawn so that the middle of the line was located at the extracellular-interface of the plasma membrane (schematic shown in Supplementary Figure S4). In this manner, a bell-curve of F-actin staining was generated. This was compiled by averaging two to five arbitrary membrane regions of interest per cell per image (Steimle et al., 2022). This analysis revealed that the shFXN (orange) trace had notable differences in membranous F-actin distribution compared to the EVEC controls (black) (Figure 6D). The membrane peak is indicated by the dotted line drawn through the curve, which shows that shFXN hBMVEC have 12.5% less F-actin at the start of the cell membrane (Figure 6E).

As mentioned, the CAR is also essential to barrier integrity by providing structural integrity, adhesion, and junctional support. CARs can span 10–300 nm below the membrane surface; thus, 300 nm regions flanking the membrane peak are represented in the gray shaded region (Figure 6D) (Belvitch et al., 2018; Svitkina, 2020; Bayir and Sendemir, 2021). The quantified CAR values are maximized in the inset, and showed that shFXN hBMVEC have significantly less F-actin in 5 of the 6 collected CAR datapoints, ranging from ∼10–15% less than the EVEC controls (Figure 6D).

We questioned if the changes in F-actin of shFXN were downstream of deficient β-actin transcriptional and translational processing. To assess this, we used qPCR, comparing β-actin to the housekeeping control B2M and found no transcriptional changes between the EVEC and shFXN hBMVEC (Supplementary Figure S5A). Western blot analysis of β-actin against total protein also showed no significant difference among the shFXN hBMVEC (Supplementary Figure S5B). Thus, we observed a significant decrease in *filamentous* actin in shFXN at the whole-cell level, at the cell membrane, and in the cortical actin ring without changes in actin transcriptional and translational processing. This indicates that the cytoskeleton is post-translationally altered downstream of FXN-loss, and that physiological functions relying on the actin cytoskeleton likely are affected in disease.

### shFXN hBMVEC have decreased tight junction abundance

**FIGURE 7:**
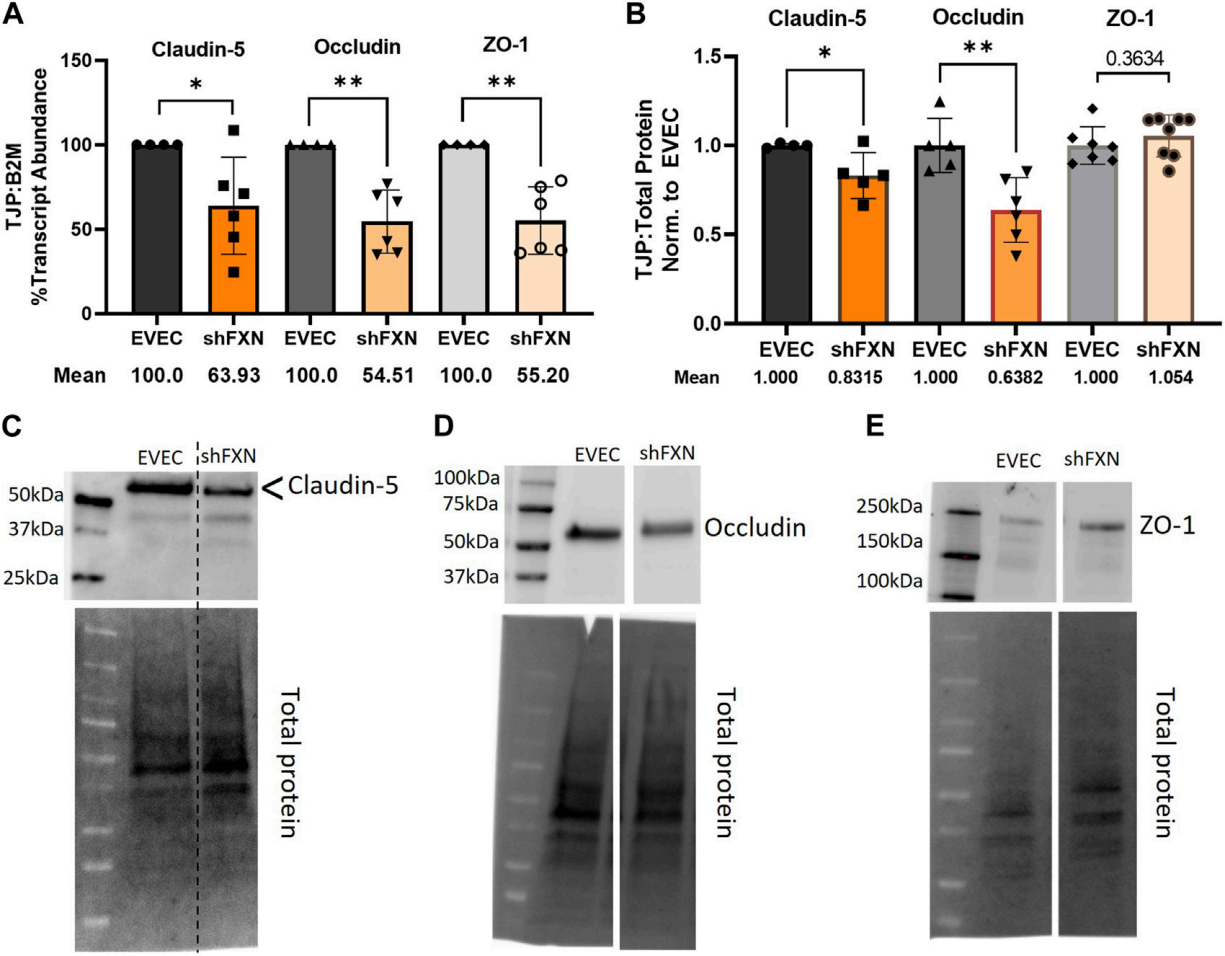
shFXN hBMVEC have decreased levels of tight junction proteins. **(A)** RNA was reverse transcribed and amplified for Claudin-5, Occludin, and ZO-1 against B2M as a housekeeping control. Transcript abundance was quantified using the ΔΔCt method normalized to the empty vector endothelial cells (EVEC). **(B)** Total protein lysates were run on a 4%–20% stain free gel, transferred to PVDF, and probed for Claudin-5, Occludin, and ZO-1 against total protein. Representative blots are shown for **(C)** Claudin-5, **(D)** Occludin, and **(E)** ZO-1. Student’s *t*-test α = 0.05, *p < 0.05, **p < 0.01. **(A)** EVEC; *n* = 4. shFXN; *n* = 6, **(B)** EVEC; *n* = 4–6. shFXN; *n* = 5–8.

Actin, and the structure of the CAR are essential in anchoring ZO-1 for the integrity of tight junction structure. Thus we assessed the presence of tight junction proteins in our model (Fanning et al., 1998; Odenwald et al., 2017; Van Itallie et al., 2017). Claudin-5 is the major claudin isoform present in brain endothelial cells (Greene et al., 2019), and so we quantified its transcription along with the tight junction protein occludin, and the scaffolding protein ZO-1 with respect to the β2M housekeeping gene. Indeed, there was significant transcriptional deficit with respect to all three tight junction proteins, with shFXN retaining only 64% Claudin-5, 55% Occludin, and 55% ZO-1 transcript, respectively (Figure 7A).

We furthermore sought to quantify protein levels of each tight junction protein, normalizing the densitometric values of each band to that of total protein. Indeed, there was a significant reduction in the transmembrane proteins Claudin-5 at 83% expression and Occludin at ∼64% expression (Figure 7B). Note that the Claudin-5 band observed is at twice the reported molecular weight (23 kDa *versus* our major band product at ∼55kDa, as identified by the arrowhead). This may be due to blotting conditions displaying our product as a dimer of the low-molecular weight protein. To validate the observed higher molecular weight band, we blotted our samples along with Claudin-5 overexpression HEK293T lysate and control HEK293T lysate (Supplementary Figure S6). Note that HEK293T cells do not inherently express Claudin-5, so the non-transfected sample serves to show non-specific bands. Indeed, the overexpression lysate (CLN5-OE) shows a band at 55 kDa (yellow arrows), and only faint bands are shown in the ∼20–50 kDa range in the control lysate (HEK). In contrast, we did not see any difference in the protein content of the cytosolic scaffolding protein ZO-1. Overall, these findings led us to examine the integrity of barrier formation by shFXN hBMVEC.

### shFXN hBMVEC have reduced transendothelial electrical resistance

**FIGURE 8:**
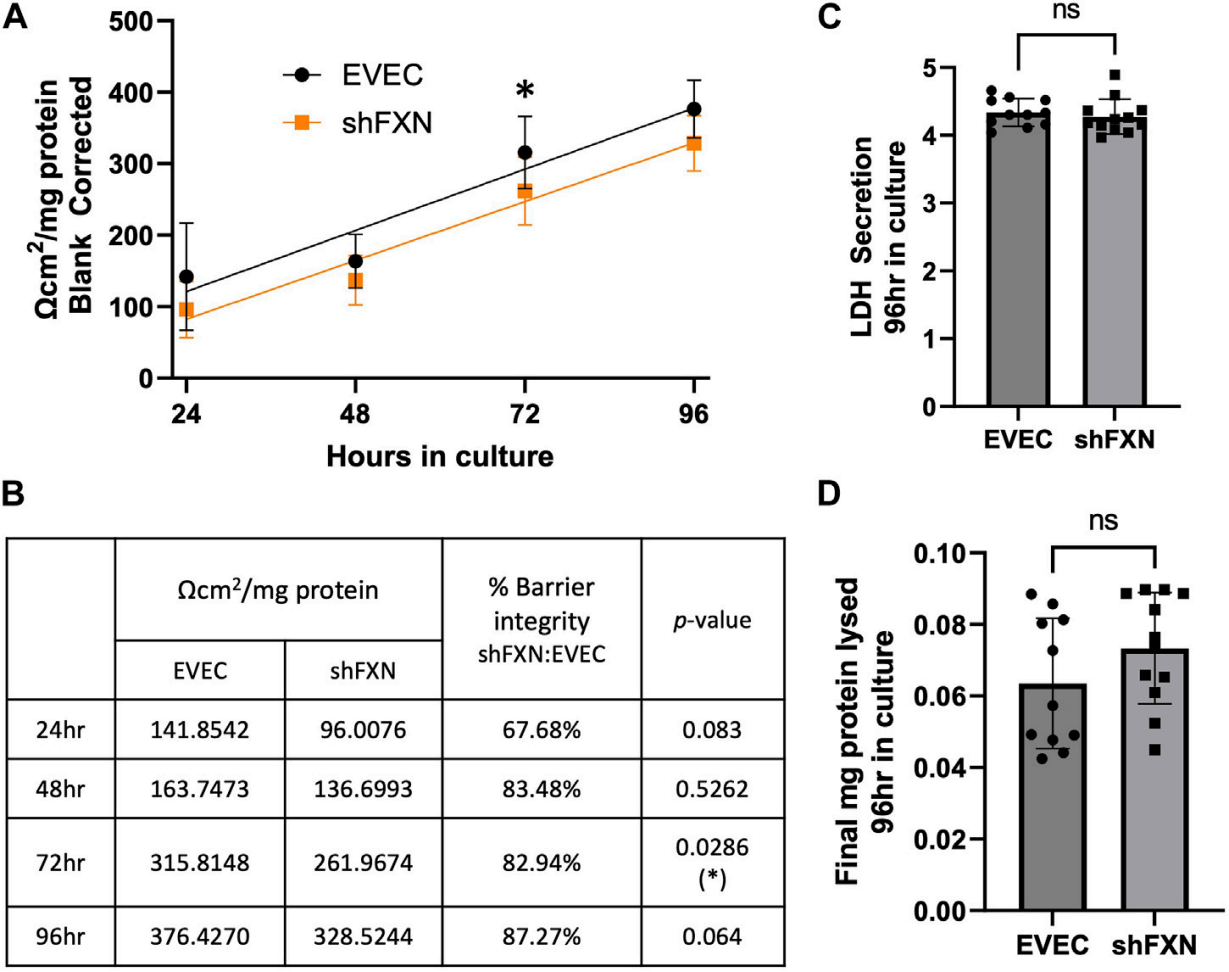
shFXN hBMVEC display a leaky barrier early on that does not reach proper resistance barriers of EVEC controls. hBMVEC plated in the apical chamber of transwells and polarized at 8-h in culture. **(A)** Transendothelial electrical resistance (TEER) measurements are taken at 24, 48, 72, and 96-h in culture. Sample wells are background corrected to a cell-free transwell, multiplied by the growth area of the 24-well transwell (0.336 cm^2^), and normalized to the final protein content of the well. A linear regression is fitted to the line and the slope values compared for similarity using the Student’s *t*-test. **(B)** Mean Ω*cm^2^/mg protein, % barrier integrity, and the *p*-value are represented at each timepoint. **(C)** LDH secretion is measured at the final timepoint to assess cell death. **(D)** Transwells are lysed at the end of the experiment and analyzed for protein content using the BCA method. **(A, B)** Two-way ANOVA α = 0.05, **(C, D)** Student’s t-test, both at a confidence interval of α = 0.05, *p < 0.05. **(A, B)** EVEC; *n* = 11 and shFXN; *n* = 12.

Thus, we sought to determine if the changes in shFXN cytoskeletal architecture (Figure 7) translated to defective barrier formation. To do so, we plated hBMVEC on mesh transwell membranes which facilitate solute flux between two chambers as a model of a cell barrier. hBMVEC were plated on the apical side of the membrane, and then polarized at 8-h in culture using serum-free growth media in the basal chamber. At 24, 48, 72, and 96-h in culture, hBMVEC were assessed for transendothelial electrical resistance (TEER) (Figures 8A, B). At the end of the experiment, apical media samples were taken to assess cell death by lactate dehydrogenase (LDH) secretion (Figure 8C). To account for any discrepancies in initial seeding density, each transwell was lysed at the final timepoint and assessed for protein content, which was used as a normalization factor (Figure 8D).

At each of the timepoints, there was a clear deficit in the shFXN (orange) barrier integrity as measured by TEER compared to EVEC (black), with barrier strength starting only at 67% (Figures 8A, B). Note that this deficit in barrier capacity is statistically significant at 72-h in culture only, but the trend of increased permeability in the shFXN barrier was observed at each timepoint. In addition, the shFXN cells approached the barrier strength of the EVEC control at later timepoints, but they never reached the resistance values of the control barrier. This likely reflects the production deficit in the tight junction-forming proteins (Figure 7).

### shFXN hBMVEC have increased paracellular tracer flux

Lucifer yellow (LY) is a cell-impermeant fluorescent paracellular tracer of ∼522 kDa. hBMVEC were plated as above and using the same time course as above were incubated with 50 μM LY in the apical chamber for 45-min. Basal aliquots were removed at the end of the incubation periods for analysis of flux. Readouts were compared to a standard curve to determine the concentration of LY fluxed per timepoint.

**FIGURE 9:**
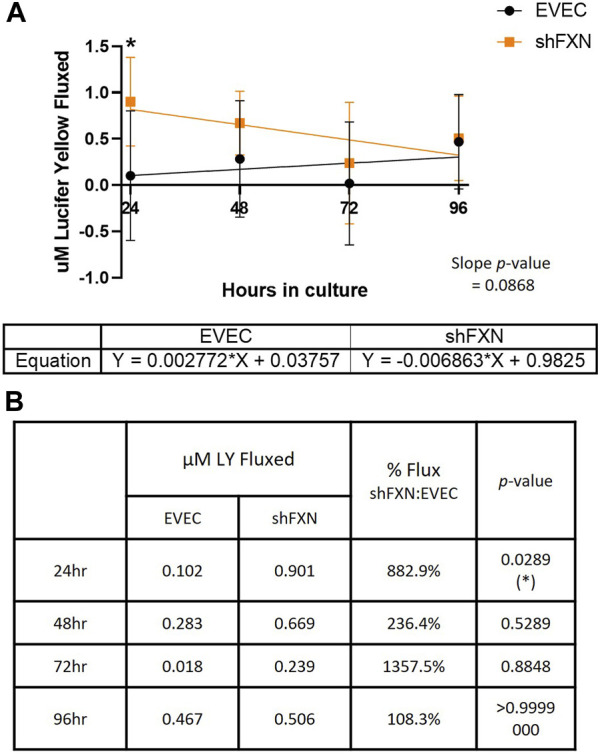
shFXN hBMVEC are paracellularly permeable to Lucifer Yellow compared to EVEC controls. hBMVEC are plated on transwells as described. At 24-, 48-, 72-, and 96-h post-plating, 50 μM of Lucifer Yellow is incubated in the apical chamber for 45-min. **(A)** Basal media aliquots are analyzed for nanomoles of LY fluxed at 24–96 h in culture at 490 nm and 525 nm excitation and emission, respectively. The cell monolayers were lysed after the final timepoint, and total protein content was used for normalization of all experiments. A linear regression is fitted to the line and the slope values compared for similarity using the Student’s *t*-test. **(B)** Mean μM LY/mg protein and the p-value are represented at each timepoint. Two-way ANOVA, α = 0.05; *p < 0.05. EVEC and shFXN; *n* = 9.

We found that shFXN hBMVEC did indeed flux increased amounts of LY in this transwell culture (Figures 9A, B), which correlated to the deficit of TEER values at these same time points (Figures 8A, B). The difference in flux started at over 800%, a statistically significant increase, but ends at nearly the same level of EVEC controls (Figure 9B). Despite these large changes, a difference in rate of flux (represented by the slope of the linear regression) did not reach significance in comparison to the EVEC controls. However, the data again reflected a time-dependent deficit in barrier formation, exhibiting a permeability deficit that did not recover over time (Figure 8A, 9A). Indeed, the negative slope of the shFXN regression indicated that the barrier continued to seal during these experiments, in contrast to the initially fully sealed EVEC barriers.

“Apparent” permeability could result from any one of the following: 1) true paracellular permeability, 2) cell death creating holes in the cell monolayer, or 3) slower growth kinetics of the shFXN hBMVEC cells. Note, however, we found no change in cell death in these transwell experiments as represented by LDH secretion (Figure 8C).

To quantify the rate of cell proliferation, we incubated *developing* cell monolayers with the nuclear dye CYQUANT Red (Supplementary Figure S7). Note that this differs from our transwell experiments in that the cells did not start off confluent, so that we could see rate of change of cell number. The 48, 72, and 96-h timepoints were normalized to the 24-h timepoint to account for any possible discrepancies in seeding density. A linear regression was fitted to the linear portion of the growth curve showed that the shFXN (orange) and EVEC hBMVEC (black) proliferated at statistically similar rates. In summary, shFXN hBMVEC were not deficit in cell growth kinetics, indicating that the lack of barrier formation in shFXN was not due to changes in proliferation. We therefore hypothesize that shFXN hBMVEC lack “building materials,” AKA tight junction proteins and cytoskeletal proteins (Figures 6, 7) for barrier formation, and not the capacity to form the barrier (Figures 8A, 9A).

To address if seeding differences or cell death events left holes in the cell monolayer, we analyzed the distribution of both EVEC and shFXN hBMVECs in transwells across the same timecourse. Transwells were fixed and then stained for cell membranes with Wheat Germ Agglutinin-Alexa647. Mounted transwells were imaged on the Lecia SP8 Confocal at ×20 magnification to acquire a large field of view suitable for examining cell coverage across the transwell membrane. Because the transwells were not flat, a 30-slice Z-stack was acquired and merged into one image (Supplementary Figure S8). Areas representing poor cell distribution in the early timepoints are demarcated by yellow dashed circles. Notably, these phenomena were only seen in the earliest timepoint, and likely reflected the large variability in TEER and Lucifer Yellow flux data at 24-h in transwell culture (Figures 8A, 9A). Nonetheless, these images, in conjunction with TEER and paracellular flux data reveal the *development* of a barrier system, which is delayed in shFXN hBMVEC (Figures 8, 9).

**FIGURE 10:**
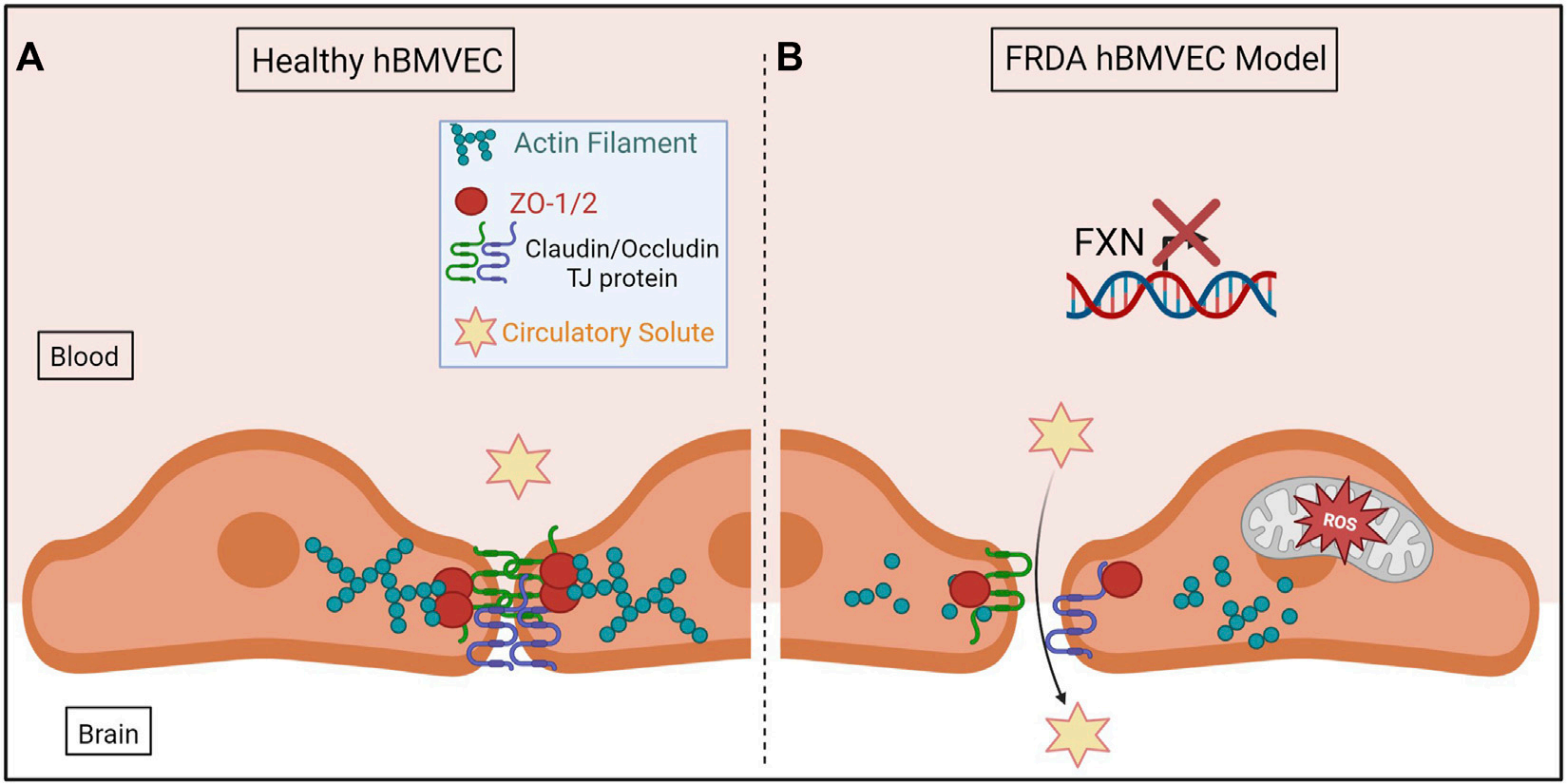
The proposed schematic of FXN-mediated hBMVEC permeability. **(A)** Healthy hBMVEC maintain FXN processing, have healthy mitochondrial dynamics, and proper actin polymerization to maintain paracellular tight junctions that prevent paracellular flux. **(B)** FRDA hBMVEC lose FXN transcriptional processing, lack healthy mitochondrial physiology, and have less polymerized actin at the membrane, leading to unsupported tight junctions and paracellular permeability.

In conclusion, we have shown for the first time that shFXN hBMVEC lost the normal organization of filamentous actin and normal tight junction processing, consistent with increased paracellular permeability (Figure 10). This cellular pathophysiology may very well contribute to brain pathology in FRDA patients, and thus represent a target for therapeutic intervention in this disease.

## Discussion

FRDA patient pathology, *in vitro* animal models, and *in vitro* biochemical data are suggestive of BBB breakdown due to loss of FXN, but this premise has not been explicitly interrogated. The data presented here may be relevant to the neuroinflammation, brain iron accumulation, neurodegeneration, and stroke experienced by FRDA patients.

Notably, brain iron accumulation in FRDA follows neurodegeneration but is progressive, suggesting that brain iron deposition occurs throughout disease (Ward et al., 2019). Similarly, cerebellar sodium content is increased in FRDA patients outside of significant atrophy, something strongly associated with development of edema (Betz et al., 1994; Ward et al., 2019; Krahe et al., 2022). Increased brain iron accumulation is co-incident with BBB breakdown in other disease models including cerebral autosomal dominant arteriopathy with subcortical infarcts and leukoencephalopathy (CADASIL) and normal aging (Uchida et al., 2020; Mezzanotte et al., 2021). Clinically, 20% of FRDA patients experience stroke, further compounding the possibility for BBB breakdown (Tsou et al., 2011; Parkinson et al., 2013; Cook and Giunti, 2017).

Molecularly, the inflammatory cytokine IL-6 is upregulated in FRDA circulation, a pathway which in neurodegeneration is linked to decreased protein expression of occludin and cadherin (an adherens junction protein), causing BBB breakdown (Cohen et al., 2013; Alsaffar et al., 2018; Khan et al., 2021; Yang et al., 2022). IL-6 also activates the M1 inflammatory microglial phenotype, which is observed in FRDA, and both contribute to BBB-breakdown and hyperpermeability in other models of neurodegeneration (Haruwaka et al., 2019; Khan et al., 2021; Vicente-Acosta et al., 2022). Further compounding evidence for brain iron accumulation via *paracellular* rather than *transcellular* flux is indirect downregulation of the iron exporter FPN by IL-6 via induced expression of hepcidin (Nemeth et al., 2004a; Nemeth et al., 2004b; Cohen et al., 2013; Alsaffar et al., 2018; Yang et al., 2022).

In line with inflammation, barrier dysfunction is also intimately linked to loss of Nrf2 transcription factor function as seen in brain, intestine, and lung (Zhao et al., 2007; Wen et al., 2019; Guo et al., 2021). Related to brain vasculature, siRNA-mediated knockdown of Nrf2 in bEnd.3, a mouse BMVEC line, and inflammatory insult caused by LPS treatment decreased transcriptional profiles of ZO-1 and occludin consistent with increased barrier permeability (Liu et al., 2021).

Furthermore, matrix metalloprotease-9 is upregulated in FRDA patient blood samples and the KIKO FRDA mouse model, potentially disrupting the integrity of the vascular basement membrane (Nachun et al., 2018; Zhao et al., 2020). Cyclooxygenases are increased in several FRDA mouse models and isolated FRDA patient B-lymphocytes, and VEGF is increased in FRDA patient olfactory mucosal mesenchymal stem cells; both pathways are correlated to downregulation of claudin-5 and occludin, the former protein being the essential claudin isoform in brain vasculature (Chen et al., 2009; Chiu and Lai, 2013; Greene et al., 2019; Pérez-Luz et al., 2020; Zhao et al., 2022). Therefore, with all aforementioned clinical and molecular details describing the potential for vascular breakdown, we sought to directly interrogate brain-derived vascular barrier integrity in shFXN hBMVEC.

Our FXN-knockdown hBMVEC model system provides a platform to examine the role brain vascular homeostasis plays in the cerebral pathophysiology in FRDA. Our shFXN model may be considered a mild knockdown due to the retention of 76% of protein expression in the shFXN hBMVEC compared to EVEC (Figure 1B), whereas classical FRDA patients retain on average only 30%. It should be noted that while the GAA-expansion mutation in adolescence is the most common FRDA genotype, ∼2%–5% of patients develop disease due to a single-allele point mutation (pFA), and “late onset Friedreich’s Ataxia” (LOFA) represents about 25% of the disease cohort (Sacca et al., 2011; Fearon et al., 2020). Residual FXN protein levels vary in these cases, with 33% FXN retention in pFA, and 65.6% in LOFA (Sacca et al., 2011). Therefore, while our model has a mild knockdown compared to classical FRDA and pFA, it is within the range of FXN knockdown linked to disease, and, most critically, displays FRDA cell pathologies including altered iron transporter expression (Figure 2), loss of antioxidant capacity due to decreased Nrf2 presence (Figure 3), decreased total energy production (Figure 4), aberrant mitochondrial networking (Figure 5C), and increased cell size (Figure 5E). As noted, our hBMVEC model is similar to ∼60% FXN protein expression in other shRNA neuronal models (Franco et al., 2012).

Our shRNA model also provides the opportunity to study a heterogeneous population because we chose non-clonal culturing. Not only does frataxin expression differ among FRDA patient cohorts, but also between different tissue types of an individual patient. FXN is most strongly expressed in tissue systems characterized by robust energy metabolism, including the brain, liver, gall bladder, pancreas, kidney, bladder and sex organs, and endocrine, gastrointestinal, and urinary systems (Koutnikova et al., 1997; Pontén et al., 2008). Thus, FXN functional heterogeneity is an inherent variable in FRDA research.

We were surprised to see in contrast to the iron starvation hypothesis, an upregulation of *both* TfR and FPN protein expression in shFXN hBMVEC (Figures 2C, D). Note, however, that our shFXN model can be considered a non-classical (lacking GAA expansion repeats) and mild knockdown (retention of 67% FXN protein compared to the 30% of patients), and may therefore not fully represent all clinical pathologies. FPN is largely post-translationally regulated via hepcidin which can be induced by IL-6, IRE-based translational regulation, or miR-485-3p. Since our shFXN hBMVEC model provides *in vitro* data, we did not examine the effect of the IL-6-hepcidin axis, IRE-regulation, or miRNA presence. This could explain the discrepancy of FPN abundance in our shFXN hBMVEC *versus* known FRDA models and clinical pathologies. Further investigation of FPN function in FRDA will be pursued in patient-derived iPSCs reprogrammed to hBMVEC. Such studies could quantify *transcellular* iron efflux into the brain*,* potentially additive to the *paracellular* flux analysis presented here. FPN is the only known mammalian iron exporter and is expressed in these cells, thus providing another avenue of investigation for brain iron flux in our model (McCarthy et al., 2014).

Downstream of iron handling in FRDA is a known increase in oxidative stress, which is further compounded in disease by a loss of the antioxidant transcription factor Nrf2 (D’Oria et al., 2013). Thus, we quantified the expression of some of the suppressors of the reduced oxygen species involved in the Fenton reaction, those downstream of the reaction of molecular oxygen with un-chaperoned ferrous iron. Indeed, we saw a significant decrease in Nrf2 protein and transcript in our shFXN hBMVEC (Figures 3A–C), a change seen also with respect to other antioxidant proteins. We expected the decrease in protein content (Figures 3A, B) since Nrf2 is mostly post-translationally regulated via degradation, but the transcriptional downregulation (Figure 3C) compounds the increase of oxidative stress in our shFXN model. For example, the mitochondrial superoxide dismutase, SOD2 was significantly downregulated (Figure 3H) without a change observed for the cytosolic dismutase, SOD1 (Figure 3G). This finding is consistent with the ferrous iron chaperone role FXN plays in the mitochondrial matrix space. Furthermore, concurrent with the known increase of cytosolic actin glutathionylation, we found a significant loss of transcription of the cytosolic deglutathionylating enzyme GLRX1 (Figure 3D), a change not reflected in the mitochondrial form, GLRX2 (Figure 3E). Thus, FXN loss creates mitochondrial oxidative stress which is compounded by a loss of Nrf2 and SOD2. Furthermore, the oxidative insult that this causes with respect to actin glutathionylation cannot be suppressed due to GLRX1 transcriptional deficit (Figure 3D).

Our cell model exhibits an interesting energy metabolic phenotype, with a significant loss of oxidative phosphorylation (Figures 4A, B) and a marginal increase in non-oxidative metabolism (Figure 4C). We hypothesize that FXN knockdown directly decreases oxidative metabolism due to lost production of iron-sulfur clusters of the, ETC, and therefore, glycolysis is upregulated to compensate for energy production. Indeed, this is represented by a decrease in the ratio of OCR:ECAR, which is normally 50:50, and accounts for a 7% shift in the increased use of the glycolytic pathway (Figures 4D, F). Overall, this does not account for drastic changes in total energy production (reduction of only ∼8%) because of glycolytic upregulation (Figure 4E).

Mitochondrial changes in our shFXN hBMVEC were twofold: 1) changes in energy metabolism as described, and 2) altered networking dynamics. We found that the total number of mitochondrial objects was statistically significantly increased in our shFXN hBMVEC compared to the EVEC controls (Figure 5C). However, when comparing the percentage of the cytosol that was being occupied by mitochondria, the values were similar (Figure 5D). This led us to investigate cell size, which we found was significantly increased by 17% in the shFXN hBMVEC in comparison to the EVEC controls (Figure 5E).

Increased cell size was correlated to increased actin glutathionylation in FRDA fibroblasts (Pastore et al., 2003). Since actin glutathionylation is pathologic to filament formation, we quantified the levels of F-actin in our shFXN hBMVEC model using phalloidin, which binds at actin-actin interfaces. A reduction in total cellular F-actin of ∼20% could be quantified at the whole-cell level (Figure 6C), but it was also apparent that there was reduction and disorganization of membranous F-actin in the shFXN hBMVEC compared to EVEC controls (Figures 6A, B).

Thus, we sought to quantify the intensity of F-actin staining in two regions of interest across the cell membrane; 1) the membrane peak, defined where the cell membrane starts, and 2) the cortical actin ring, a 300 nm structure that supports anchorage of transmembrane proteins and provides cell tone. We used ImageJ to create a histogram of signal intensity of F-actin staining across a 6.5 micron line, separating out our two regions of interest for comparison between EVEC and shFXN hBMVEC. We identified a significant reduction of F-actin at the membrane peak, a deficit of 12.5% (Figure 6E), and of most regions of the cortical actin ring, ranging from 10%–15% reduction in shFXN (Figure 6D inset). This aligns with the increase in actin glutathionylation in FRDA fibroblasts and the known pathologies in F-actin formation that this causes, (Dalle-Donne et al., 2003; Pastore et al., 2003). Here, we also correlated the lack of GLRX1 with decreased F-actin formation in shFXN hBMVEC (Figures 3D, 7). Note that the actin deficiency in these cells is not due to transcriptional (Supplementary Figure S5A) or translational (Supplementary Figure S5B) defects of β-actin processing, and thus appears post-translationally regulated.

Indeed, cortical actin is essential in tethering ZO-1, the scaffolding protein for the transmembrane tight junction proteins Claudin and Occludin (Fanning et al., 1998; Odenwald et al., 2017). We therefore became interested in the abundance of tight junction proteins in shFXN hBMVEC. Transcriptional abundance was significantly decreased in shFXN for each of the analyzed tight junction proteins; Claudin-5, Occludin, and ZO-1, each retaining only 55%–65% of normal transcriptional abundance (Figure 7A). We further identified that the transmembrane proteins Claudin-5 and Occludin had a reduction in protein content, to 83% and 63% of normal EVEC expression, respectively (Figures 7B–E). ZO-1 protein abundance seems to not be changed in shFXN, although we are interested in investigating both its function and proper membrane localization in future experiments. Note that these experiments were performed in a confluent monolayer rather than in a developing barrier as in the transwell experiments performed in Figures 9, 10. This is therefore a snapshot of the tight junction protein expression upon barrier formation, in contrast to longitudinal examination in the transwell experiments, in which tight junctions are known to “mature” over time, even after reaching confluence (Breslin and Yuan, 2023).

Based on the changes in cell size, altered actin dynamics, and a loss of tight junction processing in shFXN, we were interested in the paracellular barrier integrity of shFXN hBMVEC compared to the EVEC controls. First, we used TEER to measure the ion flux in between barrier formations of each cell type. Indeed, shFXN hBMVEC have significantly decreased barrier integrity, which approaches, but never achieves the strength of EVEC control barriers (Figure 8A). Importantly, changes in barrier function are not due to increased cell death, differential seeding, or decreased proliferation (Figures 8C, D; Supplementary Figures S7, S8). Combined with the decreased abundance of tight junction proteins (Figure 8), we hypothesize that shFXN hBMVEC are deficient in the building materials of a barrier, and not the capacity to form this barrier. Therefore, the formation of the barrier is delayed in shFXN hBMVEC compared to the EVEC controls.

In combination with TEER analysis of barrier strength, we incubated hBMVEC plated in transwells with the fluorescent paracellular tracer Lucifer Yellow (LY) and measured its flux into the basal chamber. Again, shFXN barriers are the most leaky to this paracellular tracer in the earliest timepoints, fluxing over 800% at 24-h in culture (Figure 9B). Again, this indicates that shFXN hBMVEC have a starting deficit in formation of a physiologically tight barrier, but have the capacity to eventually form a normal barrier.

Normal hBMVEC rely on F-actin organization to tether tight junctions and prevent aberrant solute flux between the blood and the brain interstitium (Figure 10A). This brings us to our final model; that our shFXN hBMVEC lose proper F-actin organization, are deficient in tight junction protein abundance, and are therefore susceptible to increased paracellular solute flux (Figure 10B).

Because our method of modeling FRDA is shRNA-mediated FXN knockdown, we were unable to investigate the known *direct* cytoskeletal alteration arising from cis-silencing of *PIP5K-1β* via *FXN* expansion tracts (Bayot et al., 2013). However, we do see a significant phenotype associated with FXN loss alone, indicating that FXN loss is *sufficient*, though potentially not *fully* responsible for cytoskeletal and barrier alterations in FRDA. Overall, our data indicate loss of blood-brain barrier integrity with FXN loss *in vitro,* thus supporting the examination of the microvasculature in FRDA, and other neurodegenerative disorders.

While our model shows modest relationships of normal FRDA physiology (modest FXN knockdown) (Figure 1), it should be noted that the defects in barrier physiology are great. Indeed, our shFXN hBMVEC express 63%–83% of the transmembrane tight junction proteins (Figure 7B), form only 67% of normal barrier capacity within 24-h of culture (Figure 9A), and are 800% more permeable at 24-h (Figure 9A). In conclusion, we note that while iron handling in FRDA is important, different cell types will manifest unique pathological phenotypes that should be examined. Indeed, barrier cell physiology in FRDA has not be thoroughly interrogated despite the essential function of vasculature to homeostasis. We trust these initial studies highlight the key metabolic features of hBMVEC and provide a platform for extending such studies to other barrier systems whose function are likely also at risk in FRDA.

## Conclusion

There are gross anatomy findings and molecular biochemical data which suggest vascular dysfunction in FRDA patients. We have identified a significant barrier deficit in our model of shFXN hBMVEC characterized by alterations in F-actin dynamics, loss of tight junction protein expression, and increased paracellular permeability. shFXN hBMVEC display a loss of total filamentous actin, and importantly, significant deficiency of F-actin at the cell membrane and in the cortical actin ring regions. This is consistent with decreased transcriptional processing of the tight junction proteins claudin-5, occludin, and ZO-1, along with a significant reduction in protein content of the two former transmembrane proteins. Indeed, this leads to increased barrier permeability and increased paracellular solute flux, with the earliest timepoints of barrier formation displaying the strongest pathologies without differences in the rate of change compared to EVEC controls. This has led us to conclude that shFXN hBMVEC are deficient in the architectural components of barrier formation, but not in the capacity to form a barrier. This may provide new insight into the pathology of brain vasculature in a model of Friedreich’s Ataxia. Our data provide a new understanding of BBB function in FRDA, identifying a potential therapeutic target in the neuroinflammation, neurodegeneration, brain iron accumulation, and stroke in this disease.

## Data Availability

The raw data supporting the conclusion of this article will be made available by the authors, without undue reservation.

## Abbreviations

B2M, Beta-2-Microglobulin; BBB, Blood-brain barrier; CAR, Cortical Actin Ring; CDN, Cerebellar dentate nuclei; DRG, Dorsal root ganglion; ECAR, Extracellular acidification rate; ETC, Electron transport chain; EVEC, Empty Vector Endothelial Cells; F-actin, Filamentous actin; FRDA, Friedreich’s Ataxia; FXN, Frataxin; hBMVEC, Human Brain Microvascular Endothelial Cells; ISC, Iron-sulfur cluster; LDH, Lactate dehydrogenase; LOFA, Late Onset Friedreich’s Ataxia; LY, Lucifer Yellow; OCR, Oxygen consumption rate; pFA, Point-Mutation Friedreich’s Ataxia; ROS, Reactive oxygen species; RT-qPCR, Real-Time quantitative polymerase chain reaction; TBP, TATA-binding protein; TEER, Transendothelial electrical resistance; ; TJ, Tight Junctions; WTEC, Wild-type endothelial cells; ZO, Zona occludens.
