# Supplementary material for: Loss of filamentous actin, tight junction protein expression, and paracellular barrier integrity in frataxin-deficient human brain microvascular endothelial cells—implications for blood-brain barrier physiology in Friedreich’s ataxia
Source: Front Mol Biosci. 2024 Jan 11;10:1299201. doi: 10.3389/fmolb.2023.1299201 (PMC10808331; doi:10.3389/fmolb.2023.1299201)
Supplement: Supplementary file 1 [file DataSheet1.docx]

Supplementary Material

# Supplementary Methods

**GFP examination**

Lentiviral transfection efficacy was visualized using GFP expression once puromycin-selected cultures began propagating. Brightfield, GFP, and merge images were acquired on the BioRad ZOE imager.

**Western Blotting**

Lysate (15-25 μg) was electrophoresed on a 4-20% stain-free gel (β-actin, TATA-binding protein, and Claudin-5) or on a 12% bis-tris gel (FXN). Stain-free gels were activated with UV exposure for 1-min prior to transfer to a PVDF membrane. PVDF was then UV-illuminated again for quantification of total protein, to be used as normalization. Bis-tris gels were transferred to nitrocellulose. All membranes were blocked with EveryBlot Blocking Buffer (Biorad) for 10 min at room temperature, and followed with overnight 4°C incubations with primary antibodies and 1-h room-temperature secondary antibody incubations as described in the main text.

**RT-qPCR**

RT-qPCR reactions were prepared and analyzed as described in the main text. All primers were used at 300 nM.

**Phalloidin Texas Red staining**

Staining was performed as described in the main text, representative images are shown of the line drawn for membrane analysis and the portion of this line which is representative of the cortical actin ring.

**CyQuant proliferation assay**

Proliferation kinetics were quantified using CyQuant Red per manufacturer's instructions. EVEC and shFXN hBMVEC were seeded at 5,000 cells per well of a 96-well plate and left to adhere for 24 h. At 24-, 48-, 72-, and 96-h post-plating, cells were incubated with the nuclear dye and background suppressor in growth media per manufacturer instructions for 1 h at 37°C. Cell abundance as represented by RFU was quantified with 622 nm excitation and 645 nm emission, respectively, on the Cytation5 (Biotek). Cell proliferation as a function of time was calculated by normalizing each day to the “starting” concentration of the 24 h timepoint. A linear regression equation was fit to the growth curve to determine the kinetics of cell growth of each line, and the slopes compared.

**Wheat Germ Agglutinin staining in transwells**

hBMVEC were plated and polarized in transwells as described. At 24, 48, 72, and 96-h in culture, cells were fixed for 10 min at room temperature in 3.7% Paraformaldehyde and 4% sucrose in PBS. Cells were washed thrice in PBS, and incubated in 0.5 μg/ml Wheat Germ Agglutinin-Alexa647 (ThermoFisher) and 0.7 μg/ml Hoechst for 10 min at room temperature. The transwells were then washed thrice again, the membrane excised, and mounted as described above. Images were taken on the Leica TCS SP8 confocal microscope at 20x magnification. To avoid areas of blurriness due to the non-flat surface of the transwells, 30-step Z-stacks were acquired and merged into one image.

## Tables

**Table 1.** Primer sequences used in RT-qPCR.

## 1.2 Supplementary Figures


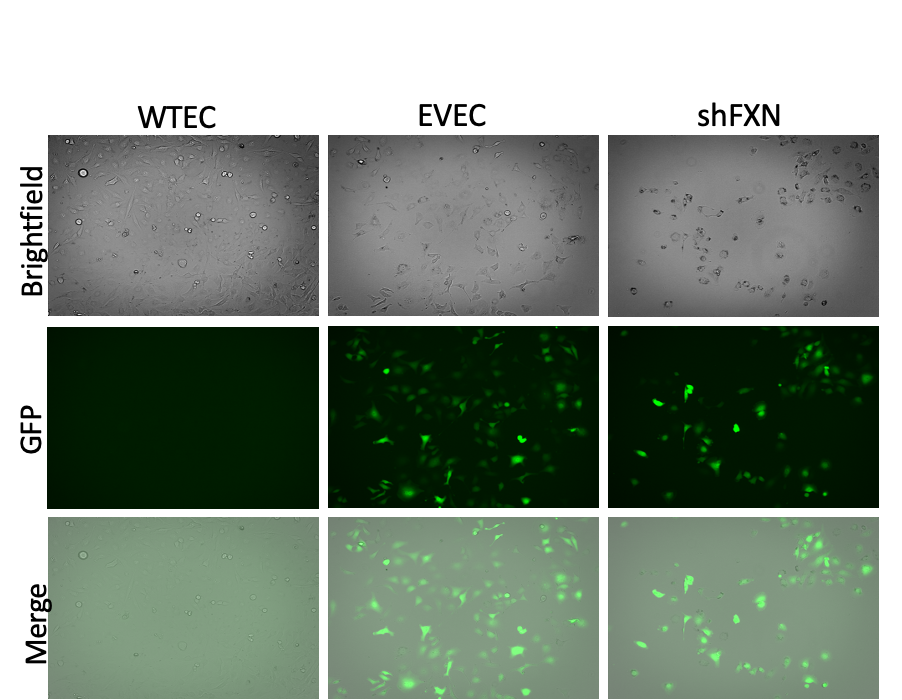


**Supplemental Figure 1**. GFP expression confirms integration of lentivirus. GFP is absent in non-transfected Wild-Type Endothelial Cells (WTEC) but present in lentiviral-transfected Empty Vector Endothelial Cells (EVEC) and shFXN.


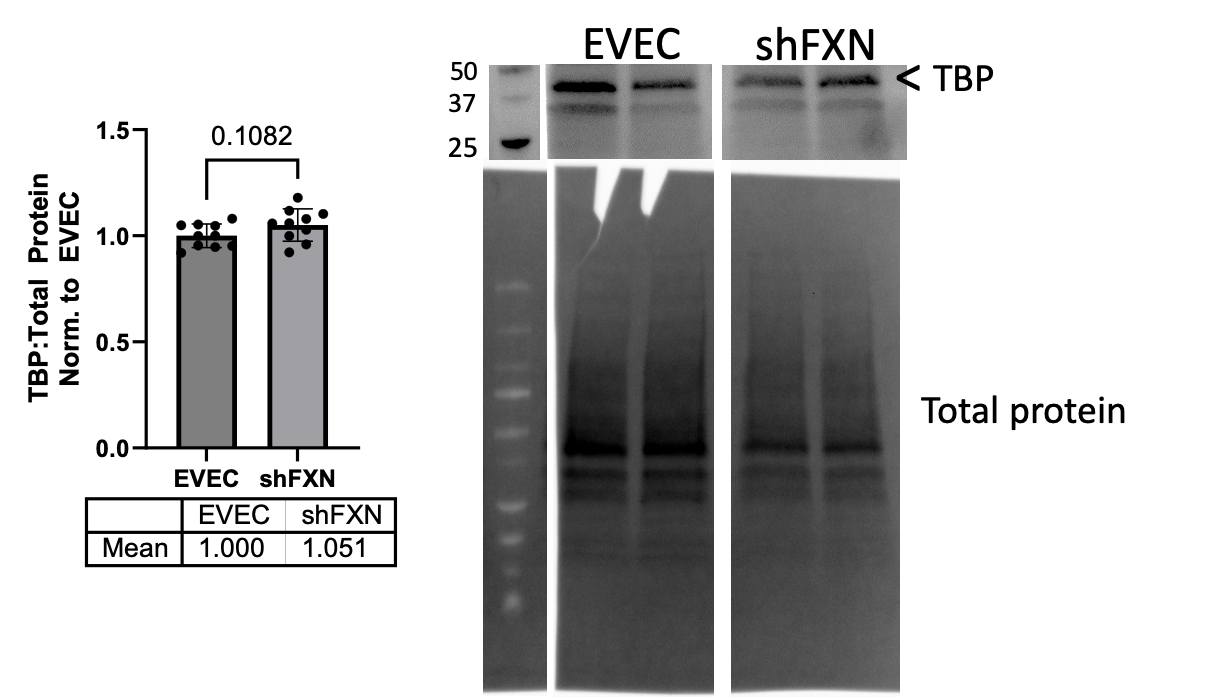


**Supplemental Figure 2**. Validation of TATA-Binding Protein (TBP) as a housekeeping gene. (A) EVEC and shFXN total lysate (20 μg) was electrophoresed on a 4-20% stain-free gel, transferred to PVDF, and probed for TBP, normalized to total protein transferred, and further normalized to EVEC controls. The 40 kDa band (identified via arrowhead) is knockout-validated. (B) Representative blot shown. EVEC and shFXN; n=10.


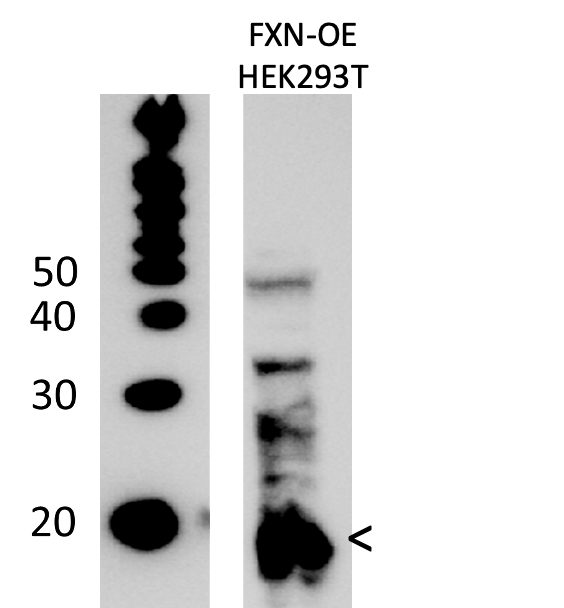


**Supplemental Figure 3.** Validation of FXN antibody. FXN-overexpression HEK293T lysate (FXN-OE, 20µg) was electrophoresed and probed for FXN for validation of bands evident with α-FXN primary antibody (Thermo Fisher PA5-13411). The ~18kDa major band designated by the arrowhead is used for protein expression analysis in our experiments.


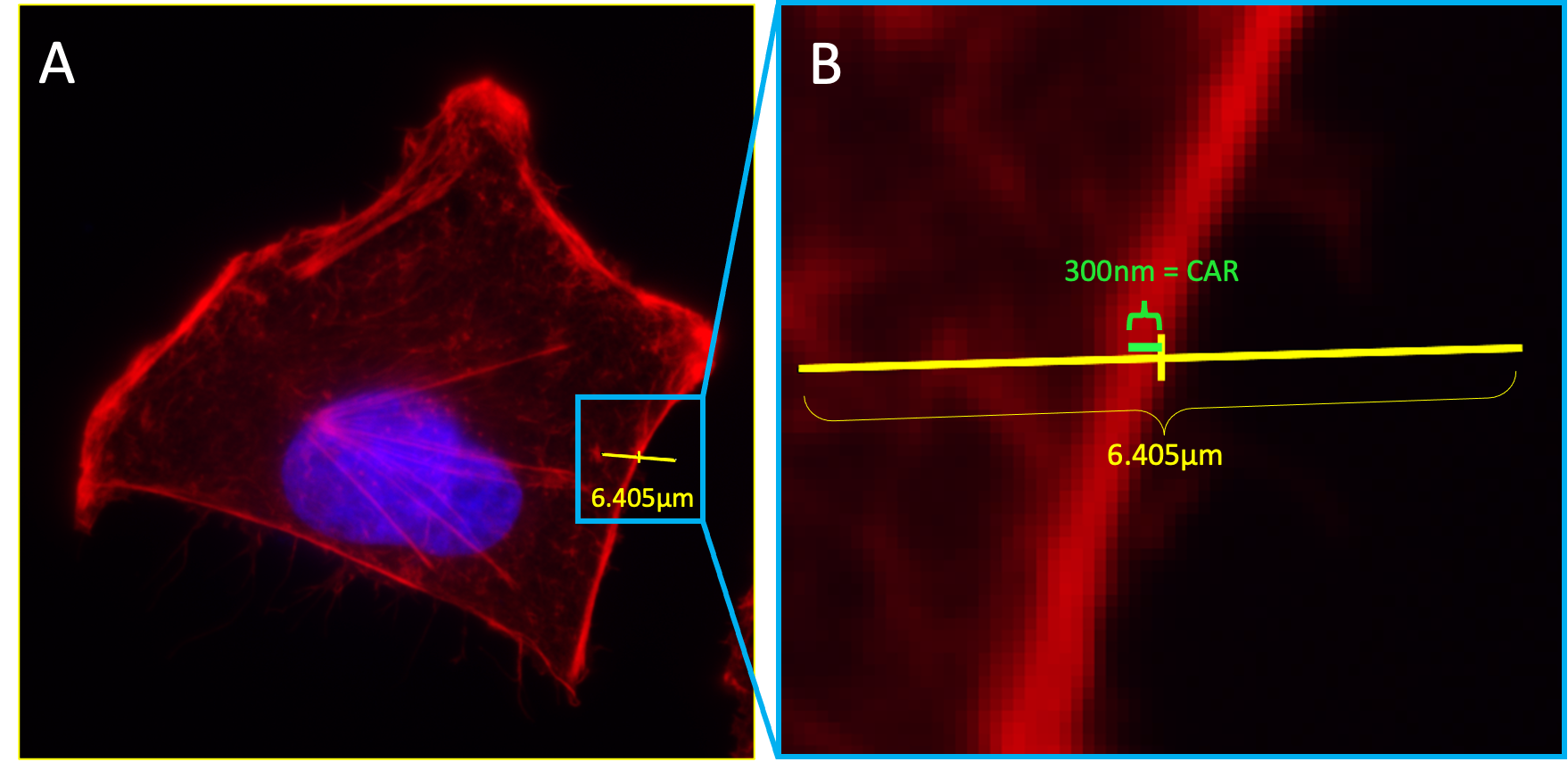


**Supplemental Figure 4.** Schematic of measurement of phalloidin line analysis and the cortical actin ring. (A) A 6.405 μm line is drawn horizontally through a cell membrane, 2-5 regions per cell. (B, inset) The 300 nm cortical actin ring (CAR) is quantified in GraphPad Prism.


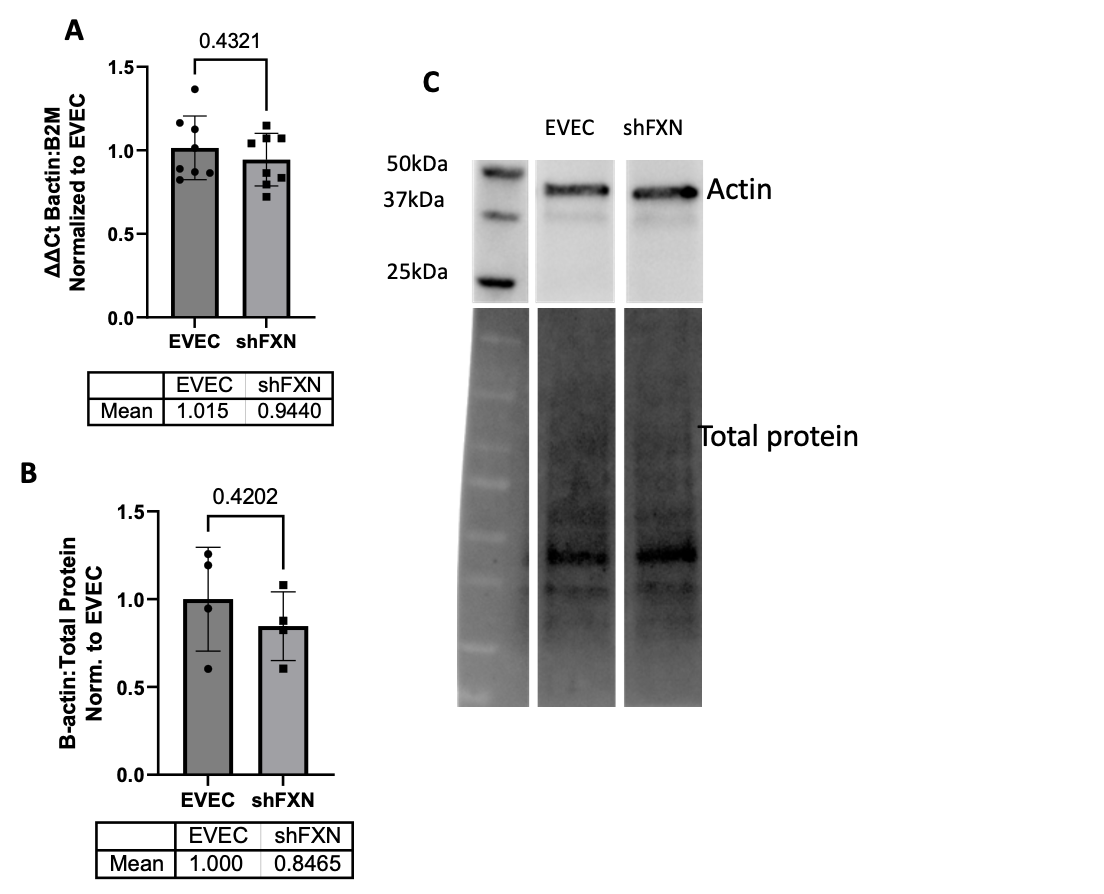


**Supplemental Figure 5.** Loss of F-actin in shFXN hBMVEC is not due to transcriptional or translational defects. (A) hBMVEC RNA was reverse transcribed and amplified for β-actin and Beta-2-microglobulin (B2M) as a housekeeping control, transcript abundance was quantified using the ΔΔCt method normalized to the empty vector endothelial cells (EVECs). (B) Total protein lysates were electrophoresed, transferred to PVDF, and quantified for total protein using stain-free technology. The blot was probed for α-β-actin and normalized to total protein. Band intensity is quantified by densitometry and further normalized to the values of the EVEC controls. (C) Representative blot shown. (A) EVEC and shFXN; n= 8. (B) EVEC and shFXN; n=4.


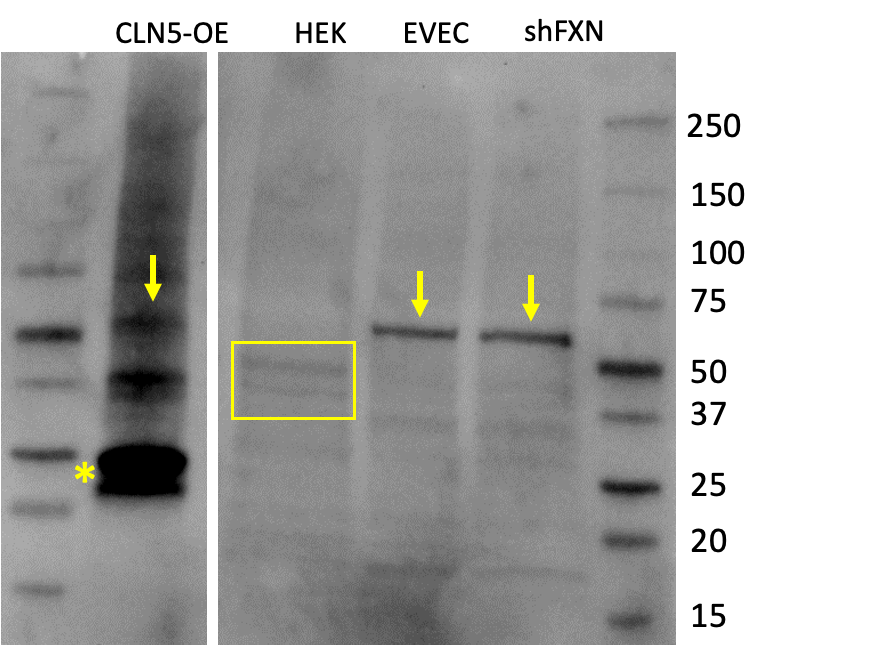


**Supplemental Figure 6. Validation of 55kDa Claudin-5 band.** Claudin-5 overexpression HEK293T lysate (CLN5-OE, 0.5 µg) was electrophoresed alongside 15 µg each of HEK293T control (HEK), and EVEC and shFXN lysates, and probed for α-claudin-5 as previously described. The major band most often quantified in the literature at ~23 kDa is visible only in the CLN5-OE (asterisk), but strong bands ~55 kDa are seen in CLN5-OE, as well as EVEC and shFXN (arrows), potentially representing dimers. Note that non-specific bands shown in HEK controls (box) are true non-specific bands, as HEK do not express claudin-5.


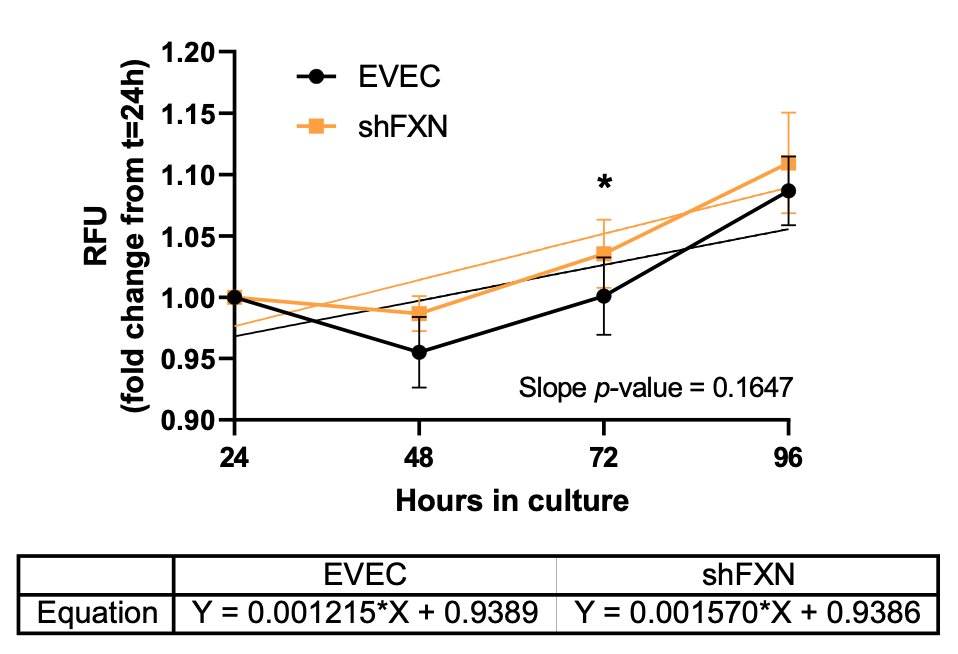


**Supplemental Figure 7.** shFXN are not proliferatively deficient. hBMVEC are plated at 5,000 cells per well and quantified for nuclear content at 24-, 48-, 72-, and 96-h post-plating using CyQuant Direct Red (ThermoFisher). All following days are normalized to the starting concentration at 24 h for a growth curve. The linear portion of the curves (48-96 h) are analyzed via linear regression (shown with dotted lines), with statistical comparison of slope values. Student’s T-test α =0.05; ns = not significant, * p < 0.05. EVEC; n= 14 and shFXN; n= 16.


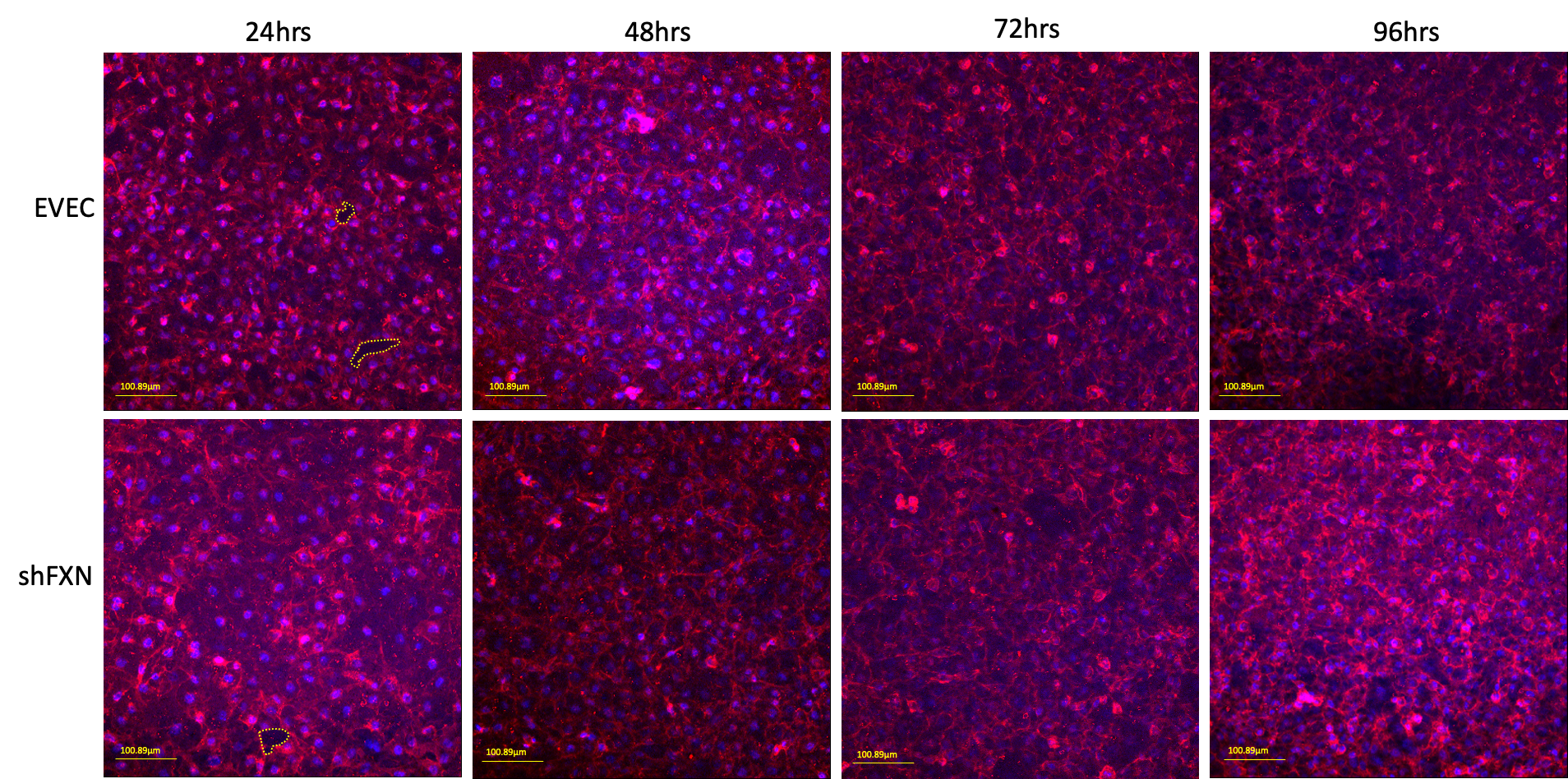


**Supplemental Figure 8.** Cell confluency on transwells in culture. EVEC and shFXN hBMVEC were seeded at 30,000 cells per transwell and polarized at 8-h post-seeding as described. At 24, 48, 72, and 96 h, membranes were stained with Wheat Germ Agglutinin-Alexa647 and Hoechst. Transwells were excised and mounted on coverslips for imaging. Images taken at 20x magnification. Areas of the membrane seeming to be lacking cell coverage in the early timepoints are circumscribed with yellow dotted circles.
